# From Pulp to Aromatic Products—Reaction Pathways of Lignin Depolymerization

**DOI:** 10.1021/acs.energyfuels.3c04509

**Published:** 2024-03-13

**Authors:** Maximilian Wörner, Alexandra Barsuhn, Thomas Zevaco, Ursel Hornung, Nicolaus Dahmen

**Affiliations:** Institute of Catalysis Research and Development (IKFT), Karlsruhe Institute of Technology (KIT), Hermann-von-Helmholtz-Platz 1, 76344 Eggenstein-Leopoldshafen, Germany

## Abstract

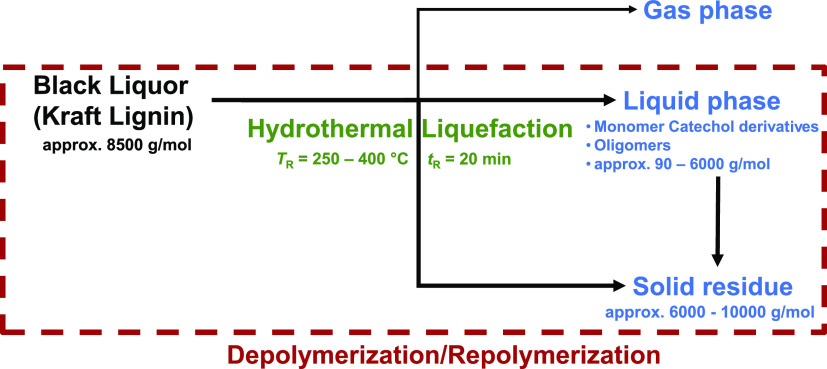

This study investigated the depolymerization of lignin into aromatic monomer compounds under hydrothermal conditions. A reaction scheme highlighting secondary alkylation reactions as well as the molecular weight shift was developed based on the experimental data. Lignin is produced in large quantities in paper production and dissolved in what is known as black liquor (BL). To avoid lignin recovery as an additional process step, BL is used directly as feedstock in the hydrothermal liquefaction (HTL) in this work. We performed various batch experiments in micro autoclaves with BL and model substances at different reaction temperatures (*T*_R_ = 250–400 °C) and a holdingtime of *t_R_* = 20 min, as well as continuous experiments (*T*_R_ = 325–375 °C, *t*_R_ = 20 min). We were able to show that different derivatives of catechols are the main products among the monomers in our process. With the help of the model substance experiments, we were able to work out three main reactions: demethoxylation, demethylation, and alkylation. This behavior could be observed in the case of BL from hardwood as well as from softwood. ^31^P nuclear magnetic resonance (NMR) spectroscopy analysis has shown that these reactions take place on aromatic monomers as well as on larger aromatic oligomer structures. At higher temperatures, a large fraction of the carbon ends up in the solid product, while the yields of the monomers decrease sharply. ^13^C NMR spectroscopy of the solid material shows that the monomers are probably incorporated into the solid phase by repolymerization. We were also able to see this effect using size exclusion chromatography analysis based on the relative molecular weight. From all of the analytical results of the products, a reaction scheme was developed that describes the reaction pathways of the lignin during HTL. Based on this, a reaction kinetic model can be developed in the next step.

## Introduction

Aromatic compounds are present all over the world, and many products are based on them, mostly petrochemical intermediates. Various commodities, like plastics, but also special chemicals and pharmaceutical products, have one or more aromatic rings incorporated in their chemical structure. Common synthetic routes start with one of the three so-called BTX substances, namely, benzene, toluene, or xylene. These are obtained as byproducts of oil refinery processes. The worldwide BTX market was evaluated at over 128 kT in 2022,^1^ most of it via catalytic reforming from the naphtha fraction of the crude oil or via steam cracking.^2^ Considering greenhouse gas emissions, about one-third of the CO_2_ emissions in the energy sector are generated from products of the oil industry.^3^ If the use of well-established aromatic chemistry is to be maintained, this will require production on a renewable basis within a zero-emission chemical industry. One option is the increased use of biomass as a feedstock. Already, many chemicals are produced on the basis of biomass-based raw materials.^4^ One possible concept for such a use of biomass is a lignocellulose biorefinery.^5−10^ The aim is to utilize the full potential for adding value to biomass as far as possible and to utilize the products both for energy and as materials. This approach also intends to ensure that such concepts are economically viable. The spectrum of biomass used ranges from specially cultivated plants such as sugar beets to various kinds of lignocellulosic biomass.^11,12^ Lignocellulose can be processed via thermochemical conversion and fractionation. The first option leads to synthesis gas, from which methanol can be produced, among other things. One possible route to produce BTX and related aromatics is the catalytic methanol to aromatics (MtA) process. There are many research studies in the field of methanol conversion to hydrocarbons. Depending on the catalyst used, the selectivity can be shifted toward aromatics.^13^ Methanol itself can be produced from biomass via various process routes.^14^ Another potentially promising material that occurs in very large quantities in nature is lignin, obtained from lignocellulose fractionation. Lignin is actually a basic component of every land plant on the earth. It is a component of the cell walls and makes a significant contribution to their stability.^15,16^ While lignin is a comparatively small component of small plants, trees can have a lignin content of more than 30 wt %.^15^ From a chemical point of view, it is a biopolymer composed of three different phenylpropanoids, namely, sinapyl alcohol, coniferyl alcohol, and *p*-coumaryl alcohol, which are connected by several different types of chemical bonds. Consequently, oxygenated aromatics can be obtained by lignin depolymerization. Therefore, the aim of this study is to observe the potential of gaining aromatic compounds from lignin depolymerization as part of a biorefinery from lignocellulose.

**Figure 1:**
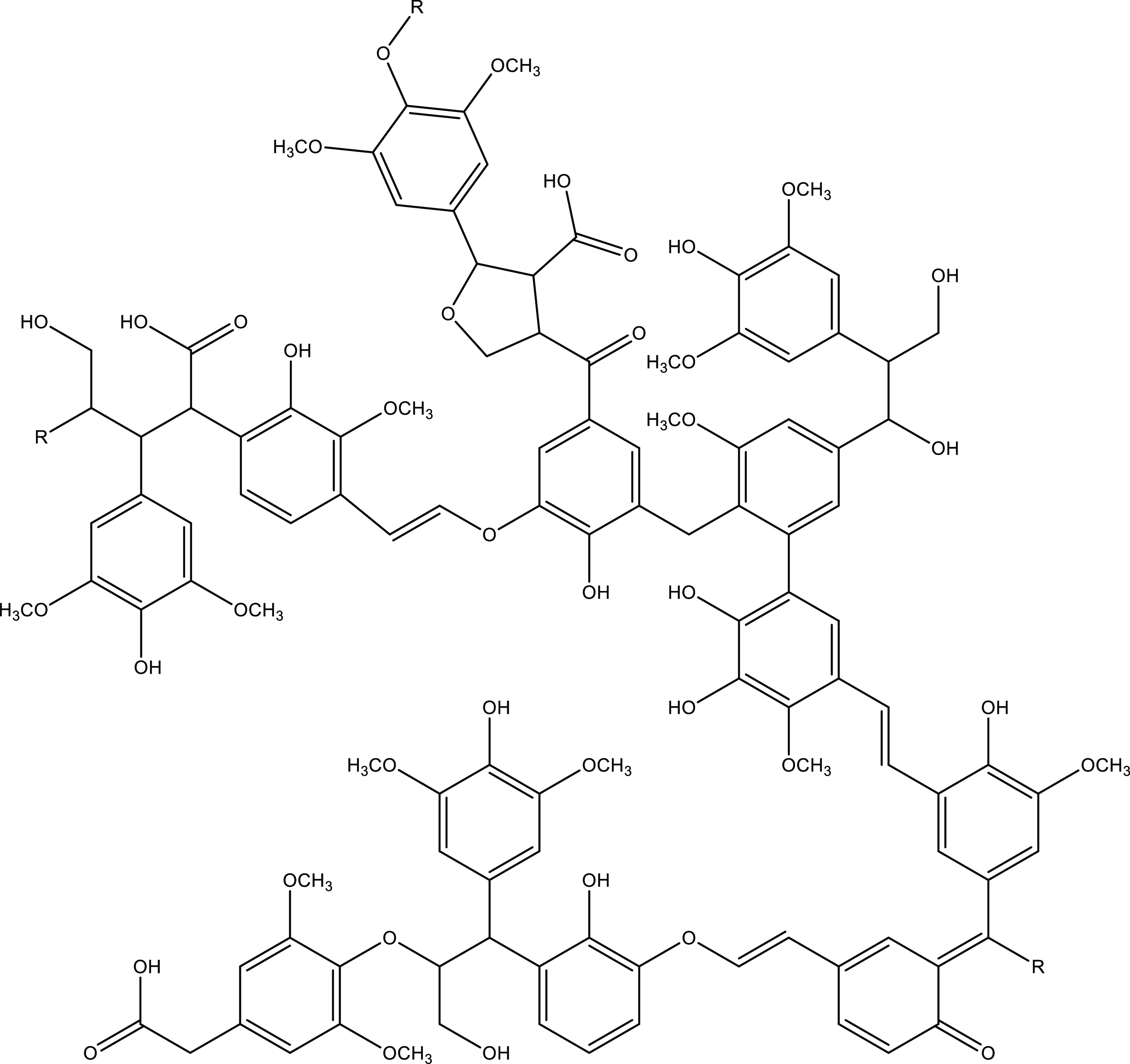
Possible chemical structure of a part of lignin; reproduced with permission from ref (18). Copyright 2013 Elsevier.

The structure of the lignin molecules differs depending on the type of plant. For example, the lignin in softwood types (e.g., pine wood) is built up almost exclusively from coniferyl alcohol (often referred to as the G-type for the guaiacyl group), whereas in hardwood types (e.g., eucalyptus wood), significantly more sinapyl alcohol (often referred to as the S-type for the syringyl group) is used as the basic building block. Overall, this results in a complex cross-linked macromolecule, as shown in Figure 1. The molecular weight ranges between 2000 and 50,000 g*mol^–1^, depending on the source and the separation process.^17^

**Figure 2:**
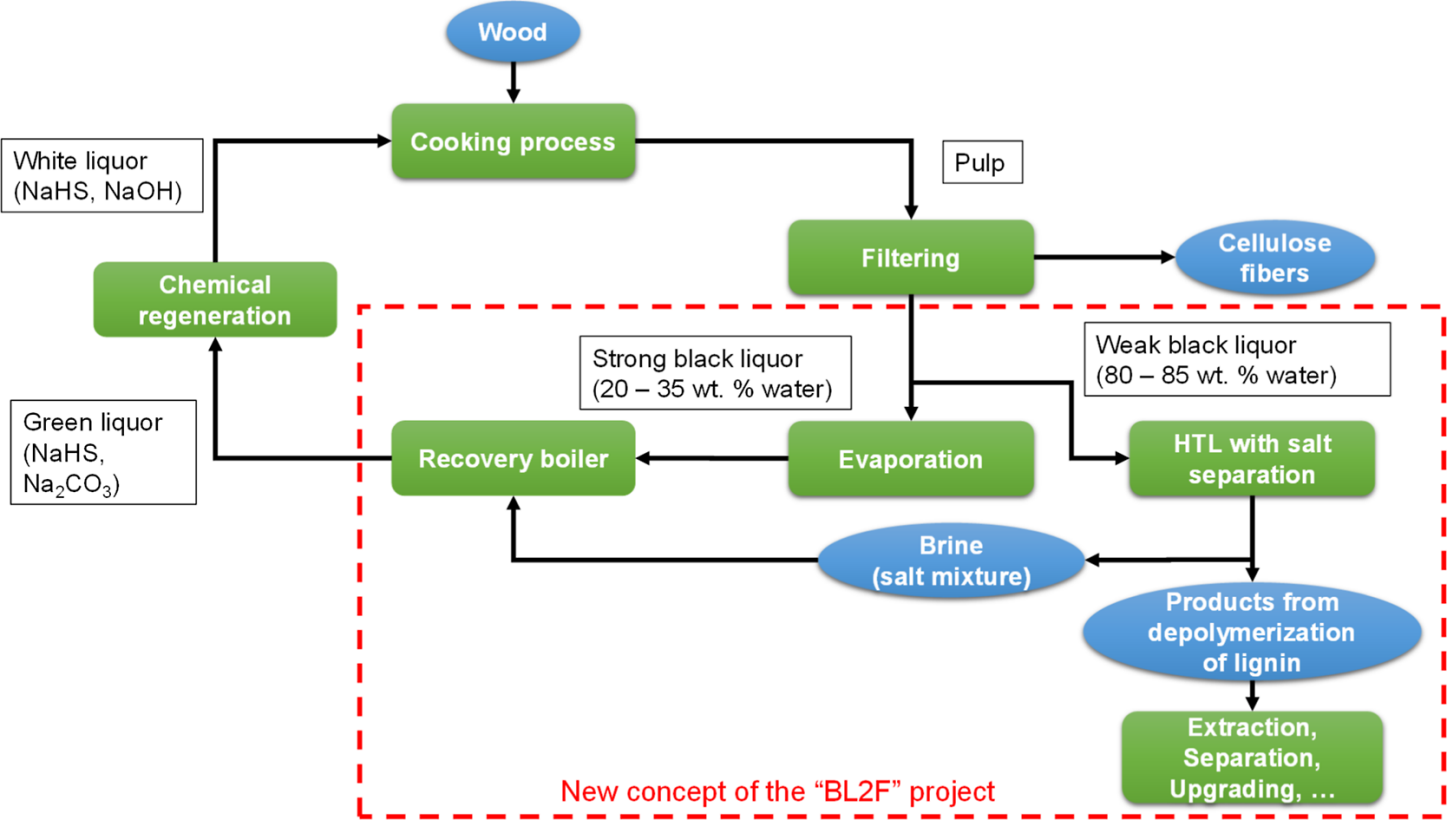
Kraft process with integrated HTL with salt separation.

Because of the high content of aromatic rings within this biopolymer, the most obvious idea is to use it to produce aromatic compounds that can be important platform chemicals or substitutes. Approximately 50 million tons of lignin are produced annually as a byproduct, almost exclusively in the pulp and paper industry.^19^ The alkaline sulfate pulping process, also called the Kraft process, is used most frequently. In this process, cellulose fibers are separated from the rest of the pulp. All of the other lignocellulose constituents, as well as the necessary cooking chemicals, end up dissolved in an alkaline solution known as black liquor (BL). The lignin structure changes at this stage since a few specific bonds can be broken during the Kraft process. The lignin produced in this way is called technical lignin. Some types of chemical bonds, such as the aryl alkyl ethers (β-O-4 bond), are already significantly reduced by the Kraft process.^20,21^ This influences subsequent depolymerization reactions. Most of the lignin produced during the pulping process is used to generate electricity and heat and to recycle the pulping chemicals (white liquor) used in this process. However, modern pulp mills produce such a large surplus of energy that it eventually has to be sold on the market for other purposes.^22^ Hence, there is also great economic potential as the production of platform chemicals may generate higher revenues with much less price fluctuation than the pure generation of “green” electricity on the free market. However, lignin recovery and further processing of BL lead to additional processing steps since the lignin must first be extracted from the BL and then dried. The EU Horizon 2020 project “BL to Fuels” (BL2F) attempts to use a new approach to process the lignin in the BL directly to produce drop-in biofuels for shipping and aircraft. The idea is to transfer the BL directly into a hydrothermal liquefaction (HTL) process. Since a water environment is required for this process, no previous treatment steps are necessary in the best case. A HTL plant, including the treatment of the products, can then be erected directly on the site of the pulp mill, for example. This would extend the biorefinery concept of a pulping mill by using lignin in the form of aromatic products.^23^ The scheme of the pulp mill concept investigated in the project is shown in Figure 2.

The complexity of the feedstock makes this goal very challenging. At the present time, the only profitable material conversion that is being carried out commercially is the production of vanillin from lignin.^24^ A lot of research work carried out over the last decades deals with the utilization of lignin, with various possibilities for the depolymerization of lignin predominating. These include basic and acid-catalyzed depolymerization, thermal pyrolysis, electrochemical, and other processes. Roy et al. have nicely summarized the different processes in their review.^25^ In addition, there are pure biotechnological approaches, e.g., to depolymerize the lignin structure with the help of enzymes.^26^ For example, Jahn et al. have studied enzymatic conversion together with electrochemical oxidation of lignin to produce aromatics of interest to the fragrance industry.^27^ Depolymerization methods based on thermochemical processes include, among others, pyrolysis and HTL.^28−32^ In their paper, Doassans-Carrère et al. compared both methods and described current research developments.^33^ As already mentioned, HTL appears to be a suitable method for the depolymerization step, utilizing the strongly changing properties of the water around its critical point (*T*_c_ = 374 °C, *p*_c_ = 221 bar) for depolymerization.^34^ Under the typical process conditions for HTL, pressures of *p* = 200–350 bar and temperatures of *T* = 250–400 °C, the water has a catalytic effect due to the sharp increase in the ionic product *K*_w_. The resulting sharp increase in H^+^ and OH^–^ concentration accelerates many acid- and base-catalyzed reactions.^35,36^ Another parameter that massively influences reactions in water under these conditions is the dielectric constant, which decreases strongly with increasing temperature and pressure.^37^ Since the dielectric constant is linked to the polarity of the substance, this leads to water behaving like a nonpolar solvent under these near-critical conditions, capable of dissolving nonpolar organic substances occurring as intermediate or final products during the depolymerization of lignin. The fact that no commercial HTL process for lignin depolymerization exists, despite many theoretical advantages, is mainly because it is not yet economically viable. This is due, on the one hand, to the chemical complexity of such processes but also to the high costs of such a process design.

Therefore, the focus of many scientific studies is to understand the depolymerization of lignin under typical HTL process conditions. Based on this knowledge, products can then be targeted in the best possible way, reaction mechanisms can be understood, and the relevant yields can be increased. In many works, technical lignin is used as a feedstock, which is dissolved in an alkaline solution. Often, potassium or sodium salts, usually as hydroxide or carbonate (NaOH, KOH, Na_2_CO_3_, and K_2_CO_3_), are used for this purpose as they can act as homogeneous catalysts. Belkheiri et al. have investigated the influence of the sodium to potassium ratio on the yields of various product phases as well as on phenolic products.^38^ It was shown that suspended solids in particular decreased in the light oil fraction with increasing sodium content, while they increased significantly in the heavy oil fraction. The difference between the two phases is in the solvent used. The Na/K ratio had hardly any influence on the other factors investigated. It is also possible to add heterogeneous catalysts. Breunig et al. used an iron–sulfur catalyst for the depolymerization of lignin based on the Bergius process for the catalytic hydrogenation of coal.^39^ Forchheim et al. worked in HTL processes with lignin and model substances using Raney nickel,^40^ which led to the acceleration of hydrodeoxygenation and thus to higher phenol yields. In both cases, however, the recovery of the catalyst turned out to be a problem. In addition to other catalysts, a solvent mixture can also be used as a reaction mixture. In the study by Cheng et al., a strong depolymerization of lignin with an original molecular weight *M*_w_ of 60,000 to 1010 g mol^–1^ was achieved in a water/ethanol mixture (volume ratio 50:50).^41^ A major drawback of the depolymerization is the simultaneously ongoing repolymerization reactions triggered by newly formed, more reactive substances that can react with each other again. Forchheim et al.^42^ and Gasson et al.^43^ considered repolymerization in their jointly developed kinetic models of HTL of lignin and solvolysis of lignin. One idea for suppressing these undesired reactions is to use capping agents like phenol or boric acid.^38,44^

Due to the complexity of the lignin molecule, the conversion leads to a range of product phases with a large number of chemical species, making it difficult to assign intermediate and final products. Therefore, Wahyudiono et al. chose catechol as a model substance and obtained phenol via a HTL process. They determined the kinetic parameters based on their experimental results.^45^ Another way to obtain kinetic data is to keep the variation of the influencing factors as small as possible and to work in special reactors. Yong and Matsumura tested the behavior of lignin under sub- and supercritical conditions in water, working with a very small reactor volume.^46,47^ This leads to a very high heating rate, which prevents intermediate and subsequent reactions during the heating and cooling processes. They were able to investigate very short reaction times of 0–10 s. Based on the results, they developed a reaction scheme with kinetic parameters and were able to show that lignin conversion follows a constant gradient in the Arrhenius plot in both the subcritical and supercritical regimes. As mentioned earlier, most research uses extracted and recovered lignin.^48^ The direct use of BL in a HTL process has been investigated by Orebom et al., with a focus on bio-oil yields.^49^ They came to the conclusion that the best yields can be achieved in a reaction temperature range of *T*_R_ = 370–380 °C. They also showed that if the dry matter content in the BL is too high, this has a negative effect on the yield.

This study aims to better understand the depolymerization of lignin in BL under hydrothermal conditions in the presence of the cooking chemicals used in the pulping step. Since BLs can differ due to the wood type as well as from one supplier to the next,^50^ it was necessary to start with a parameter study utilizing BL and relevant model compounds as feedstock. In this work, we focused on the reaction temperature due to its preponderant influence. Previous research has focused in most cases only on the monocyclic end products, as with Forchheim et al., who discussed the oligomers as a “black box”^42^ in the reaction scheme. With this work, we would like to provide a lumped reaction scheme that includes the behavior of oligomers and will be the basis of a kinetic model. To accomplish this, we investigated oligomeric products by using advanced spectroscopic methods. For this purpose, we mainly relied on 1D NMR analyses using ^13^C and ^31^P nuclei. In the field of spectroscopic characterization of lignocellulosic biomass, numerous publications have been written using either plain 1D methods with mostly ^1^H, ^13^C, and ^31^P after derivatization, or more complex 2D (^13^C and ^1^H)-correlated methods (e.g., HSQC: heteronuclear single quantum correlation). The majority of these methods are performed in solution using deuterated polar solvents (e.g., CD_3_OD or DMSO-*d*_6_) or they can also be performed on homogeneously ground solid-state samples (^13^C MAS or CP/MAS: cross-polarization magic angle spinning).^51−58^ The most relevant papers were used as references to evaluate the NMR spectra produced in the work. Together with size exclusion chromatography (SEC), we wanted to gain insight into the molecular size and functionalities of oligomers in order to setup a reaction mechanism for the depolymerization of lignin to aromatic compounds.

## Materials and Methods

The BL was provided by the Figueira da Foz pulp mill in Portugal (the Navigator Company). The wood used for the Kraft process on site came from eucalyptus plantations based on the species *Eucalyptus globulus*. This wood species belongs to the hardwood category, which leads to an S/G ratio of approximately 70:30. The BL is a malodorous dark brown to black liquid. All constituents, such as lignin or cooking chemicals, are almost completely dissolved, resulting in a strong homogeneity. The relevant properties as well as the elemental composition of the BL are listed in Tables 1 and 2. The yields in the results were calculated based on *w*_BL, burnable_. We assume that the burnable fraction is close to the organic fraction (see Cardoso et al.^50^). Differences between the burnable fraction and the real organic fraction arise due to the change in ash composition due to combustion. For comparison with BL based on softwood (spruce wood), three tests were carried out with softwood BL from Scandinavia. The properties were assumed to be equal to those of the other BL since the differences in dry matter content, pH, and density were only marginal.

**Table 1:** Properties of the BL used in the experiments; *w*_BL, burnable_ calculated from the loss on ignition corrected from the dry matter

dry matter *w*_tr_	ash content *w*_ash, 815 °C_	dry matter-based loss on ignition	raw BL-based burnable matter *w*_BL, burnable_	density ρ_BL_	pH
14.5 wt %	6.1 wt %	57.9 wt %	8.4 wt %	1.0725 kg*L^–1^	>12.5

**Table 2:** Elemental composition of the dry mass of the BL and of the extracted lignin; Analysis performed via elemental analysis (EA) and inductively coupled plasma–optical emission spectrometry (ICP–OES);oxygen calculated via difference, no other element was detected in relevant amounts

element symbol	mass fraction dry mass BL/wt %	mass fraction extracted lignin/wt %
C (EA)	34 ± 0.4	60.3 ± 0.1
H (EA)	3.4 ± 0.5	5.7 ± 0.1
N (EA)	<0.1	<0.1
S (EA)	4.7 ± 0.1	2.6 ± 0.1
O (diff.)	38.8	31
Na (ICP)	17.7 ± 0.9	0.4 ± 0.02
K (ICP)	1.3 ± 0.06	<1
sum	100	100

**Figure 3:**
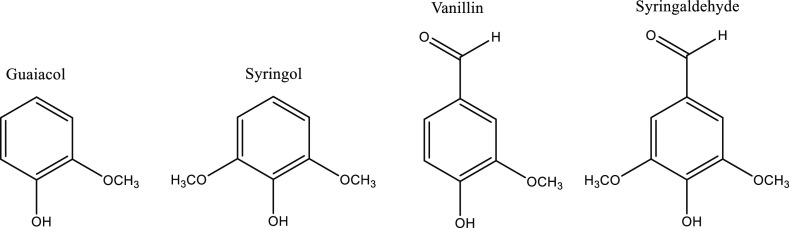
Monocyclic phenolic compounds used in experiments with model substances.

Several tests were also carried out with different model substances for the sake of comparison. These are guaiacol, syringol, vanillin, and syringaldehyde (see Figure 3). In each case, 1 g of the substance was added to 15 mL of a salt solution corresponding to a real BL. The exact composition of this solution can be found in the Supporting Information in Table S1.

### Batch Experiment Setup and Product Separation

For the batch experiments, micro autoclaves were used, which were made of stainless steel 1.4571(316Ti) in the institute’s mechanical workshop. Each of these autoclaves has a volume of *V* = 25 mL. Closing and opening of the reactors took place in a specially designed station (see Figure S1). The filling levels of BL had to be adjusted to the respective temperatures in order to avoid too large pressure fluctuations between the individual experiments. A pressure of 200–250 bar was specified as the target. The filling levels depend on the density of water at the respective temperatures and pressures^59^ (volumes used can be found in Table S2).

To achieve a fast heating rate, a fluidized sand bath (SBL 2, Techne, Stone, UK) was used. For ease of handling, it was decided to use a general heating time of *t*_pre_ = 10 min for all experiments. Studies carried out previously with the same heating procedure showed that the desired reaction temperatures were reached within 10 min. After the heating time, the holding time *t*_R_ started at the corresponding reaction temperature *T*_R_. This study investigated the reaction temperatures of *T*_R_ = 250–400 °C and holding times of *t*_R_ = 5 min (model subtances) and*tR*_r_ = 20 min (black liquor). As soon as the holding time *t*_R_ was reached, the autoclaves were cooled in a water bath in order to terminate all reactions taking place (quenching step). The gas was directed into a gas trap when the autoclaves were opened.

**Figure 4:**
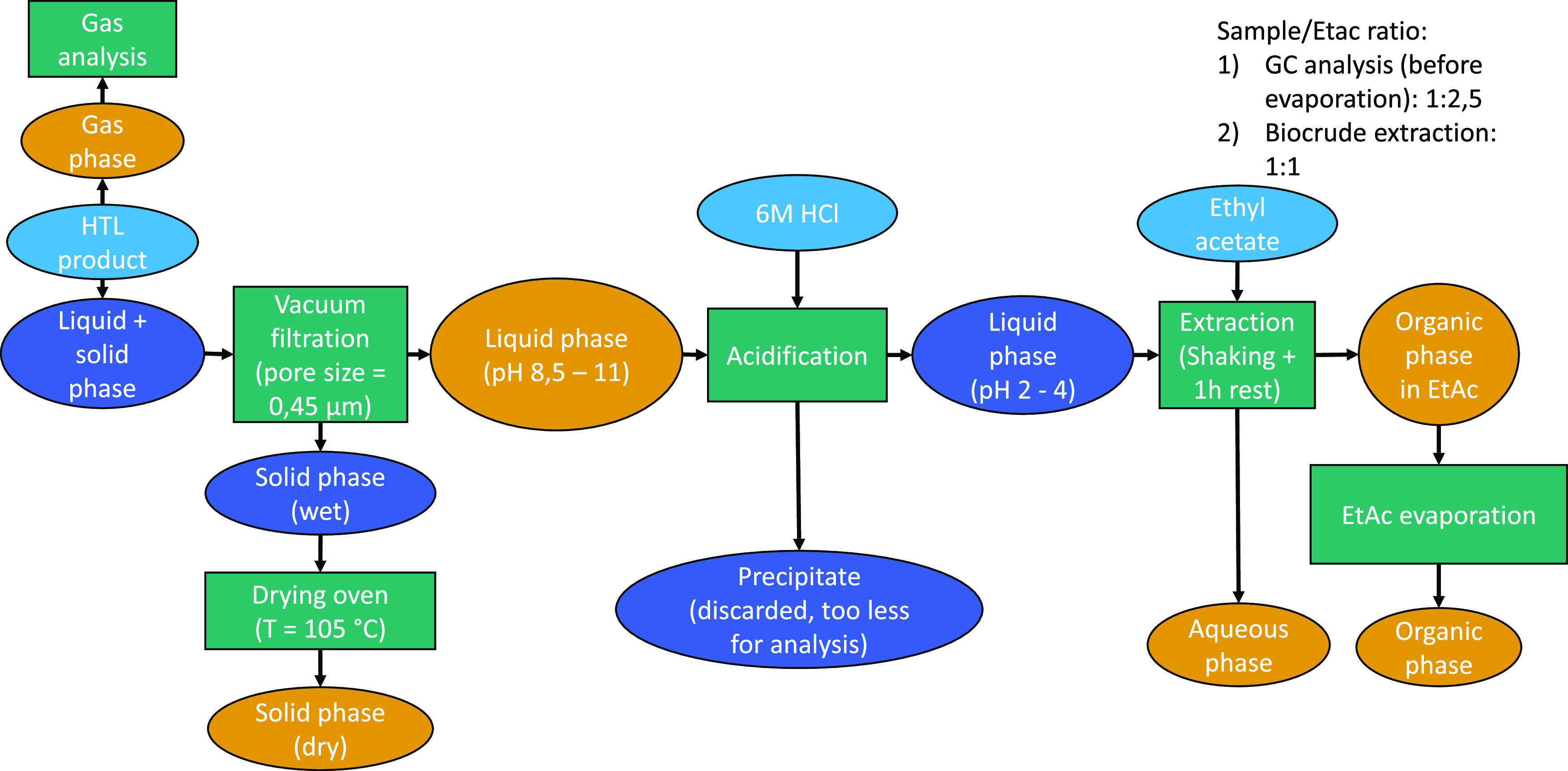
Procedure for product separation and analysis: dark blue products that are not analyzed; green: procedure steps; light blue: input streams; orange: product phases that are analyzed with at least one method; and EtAc = ethyl acetate.

Subsequently, the reactor was opened, and solids and liquids were separated by means of vacuum filtration. The filter used is made of nylon with a diameter of 47 mm and a pore size of 0.45 μm (Whatman, GE Healthcare, Buckinghamshire, UK). The accumulated solid was dried in an oven at 105 °C for at least 24 h. Liquid–liquid extraction (LLE) was performed to separate the organic phase from the aqueous phase. Two mL of the liquid phase had to be acidified with 6 M hydrochloric acid to a pH of 2–4. After filtration, 0.52 mL of ethyl acetate was added to 1.3 mL of the filtrate, which served as the extractant. After shaking, the sample was allowed to rest in the vial for 1 h to allow for complete phase separation. For larger scale extraction, three samples from the same reaction conditions were poured together, and the amount of ethyl acetate used was increased to a 1:1 ratio with respect to the liquid product. Figure 4 shows the procedure for separating the products in a flowchart.

### Continuous Experiment Setup

**Figure 5:**
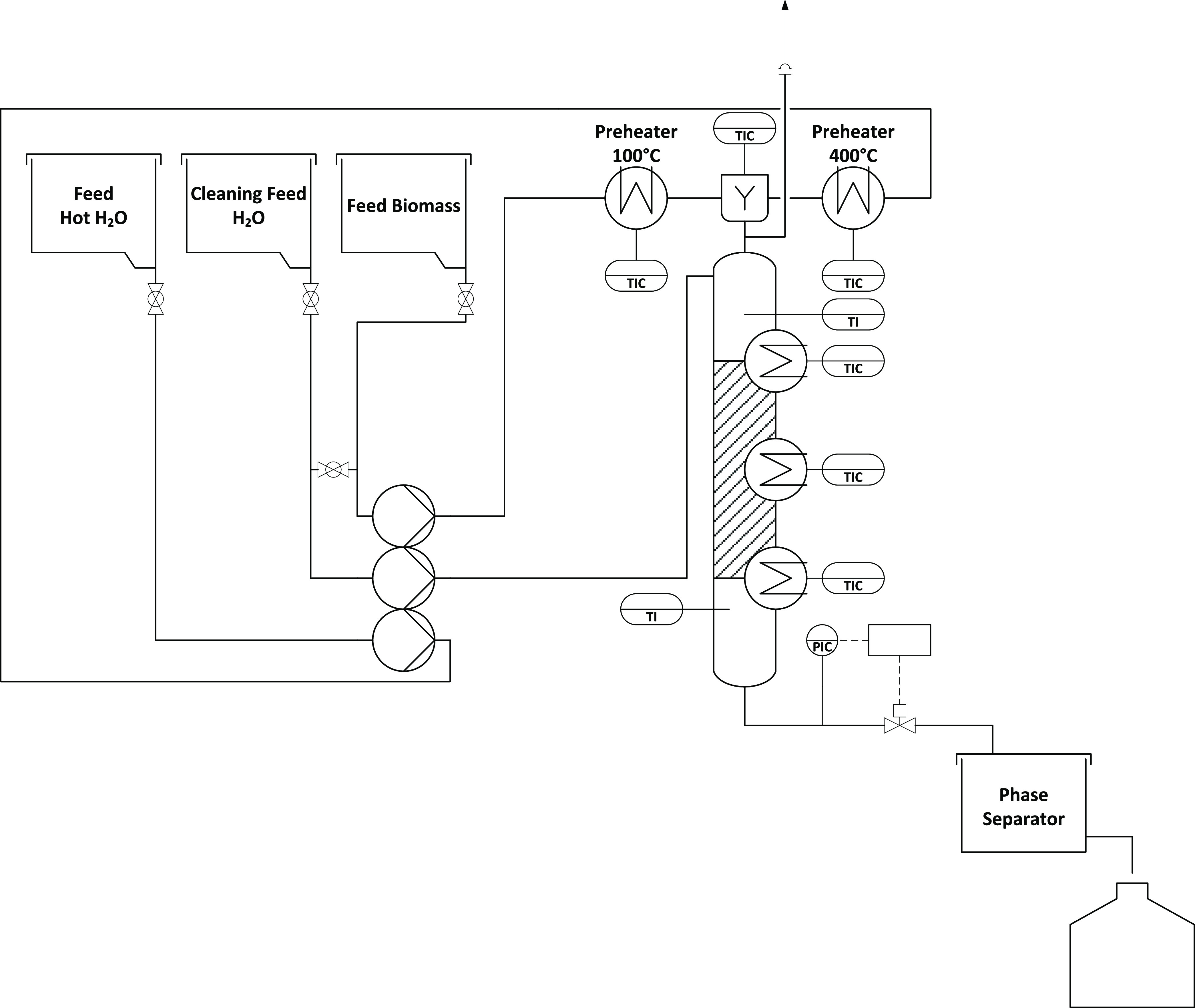
Process scheme of the continuous plant.

The continuous HTL experiments were performed in a tube reactor (Figure 5). One feed stream with hot water (*T* = 400 °C) and another with BL (*T* = 100 °C) were combined in a preheated mixing head above the reactor in a 1:1 ratio. This ensured the desired reaction temperatures *T*_R_. A three-headed diaphragm metering pump (ecoflow LDB1 diaphragm metering pump, LEWA, Leonberg, Germany) was used to supply the reactor with feedstock and process water. Heat was supplied to the reactor via three separate heating zones. After the outlet, the product stream ran into a phase separator. It was then possible to collect the products, liquid phase and solid. Three experiments were carried out at *T*_R_ = 325, 350, and 375 °C with a residence time *t*_R_ = 20 min. The system was heated with a hot water stream beforehand. When the reaction temperature *T*_R_ was reached inside the reactor, the BL stream was added. The overall feed rates were adjusted between 1.35 and 2 kg*h^–1^ depending on *T*_R_. The plant ran for one h before we started the sampling process to make sure that the stationarity of the process was reached. Product preparation followed the same scheme as that for the batch experiments.

### Analytical Procedure and Assessment

Each intermediate or final product, colored orange in Figure 4, was analyzed with at least one analytical procedure. A sample of the gas phase collected in the gas trap after opening the micro autoclaves was injected with a gastight syringe into a gas chromatograph (GC 6890, Hewlett-Packard, now Agilent, Santa Clara, CA, USA; columns Hayesep Q, Molsieve 5A, Restek, Bellefonte, PA, USA). Using a FID (flame ionization detector) and a TCD (temperature conductivity detector), it was possible to detect and quantify various permanent gases and hydrocarbons (see Table S4). The gas compounds that are detectable, as well as the calculation procedure for the carbon mass in the gas phase, are found in the Supporting Information. The dried solid was analyzed by EA (Vario EL Cube, Elementar Analysentechnik GmbH, Hanau, Germany). To complete the carbon mass balance, total organic and inorganic carbon concentrations were determined in the total liquid product prior to extraction using a Dimatoc 2100 (Dimatec Analysentechnik GmbH, Essen, Germany). Together with the mass of the collected solid as well as the collected liquid, the C mass balance could be completed.

The organic phase in ethyl acetate after LLE was analyzed for aromatic monocyclic compounds using a GC–MS (GC 6890N and 5973 MSD (mass spectrometry detector), Agilent, Santa Clara, CA, USA) and a GC-FID (GC 7820A, Agilent, Santa Clara, CA, USA). By using the GC-FID and pentadecane as an internal standard (ISTD), we were able to quantify a selection of aromatic compounds. For this purpose, the ISTD was added to the ethyl acetate in a defined amount prior to extraction. Together with the distribution coefficients *K*_i_ of the individual components (see Figure S5 in Supporting Information), this allowed us to calculate the concentration of species i in the total liquid phase β_i_ (see eq 1). Factors a–c represent the total dilution of the original sample, the ratio between the sample volume and the volume of ethyl acetate, and the ISTD factor. β_i,raw_ represents the measured concentration in the sample. The yield in relation to the biomass in the BL feed *Y*_*x*_ was calculated using eq 2 with the obtained mass for liquid product *m*_liq,prod_, and the mass of feedstock *m*_feed_
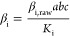
1
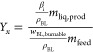
2

In addition, we quantified potential dimethylcatechols and trimethylcatechols over the areas of the peaks. The ratio of concentration to peak area of the other catechols (methylcatechols and catechols) was taken as a reference point for this. For experiments with model substances, the molar yields were determined. High-performance liquid chromatography (HPLC; Merck Hitachi Primaide, Hitachi, Tokyo, Japan; Aminex HPX87H, Bio-Rad, Feldkirchen, Germany) was used to determine acids and alcohols in the aqueous phase. After the evaporation of the ethyl acetate, the relative molecular weight of the biocrude obtained was first determined. We used SEC (LaChrom diode array detector DAD L-2455, Merck, Darmstadt, Germany), with a Viscotek A2500 column, Malvern Panalytical, Malvern, UK.

In addition to standard analytical methods, various NMR spectra were recorded using adequate pulse sequences and probe heads. The solids were analyzed using 1D ^13^C solid-state NMR. For the analysis of the liquid biocrude, we used 1D-^31^P spectra after derivatization. The procedure for the ^31^P NMR can be found in the paper published by Korntner et al.,^60^ according to the following principle: the lignin hydroxy (OH) groups were quantified by ^31^P NMR after derivatization of the lignin with a derivatization reagent. The dry lignin sample was dissolved in a mixture of pyridine, *N*,*N*-dimethylformamide, and deuterated chloroform (CDCl_3_) in the presence of an ISTD and a relaxation reagent and then phosphorylated using a solution of derivatization reagent in a mixture of pyridine and CDCl_3_. The NMR spectrum of the lignin and ISTD derivatives was then acquired using liquid NMR spectroscopy, and the OH groups were quantified by the relative integration of the corresponding signals for lignin and ISTD. These experiments were performed by BOKU—University of Natural Resources and Life Sciences, Vienna. Single pulse ^13^C MAS/CP-MAS NMR spectra were recorded under ambient conditions (1010 mbar, 20 °C) using a Jeol Spectrometer of the JNM-ECZR series, equipped with a 9.4 T Oxford Cryomagnet (resonance: ^13^C @100.51621 MHz ^1^H@ 399.90513 MHz). The solid-state spectra were recorded with a JEOL Automas solid-state probe head. More detailed information about the ^13^C NMR can be found in the Supporting Information.

## Results and Discussion

### Batch HTL Experiments with BL

**Figure 6:**
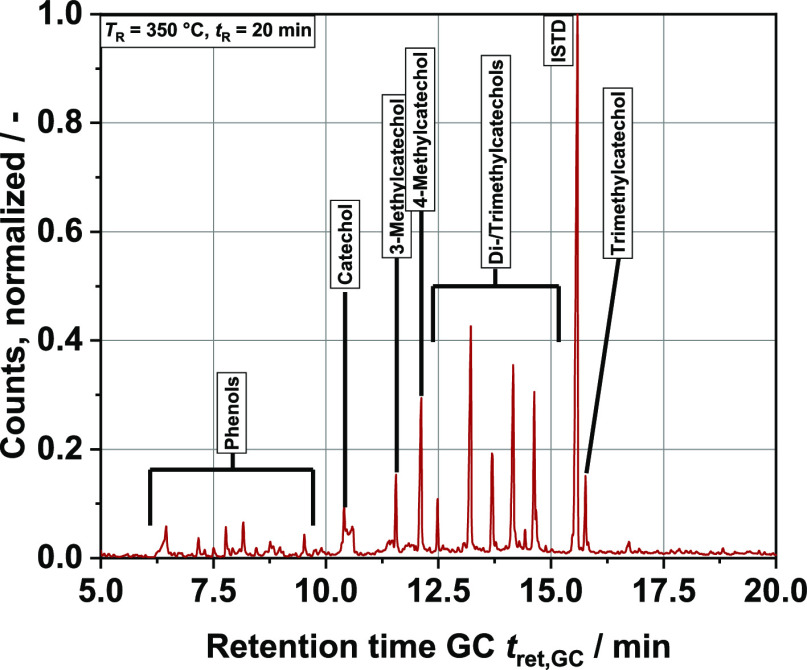
GC–MS spectrum of an extracted organic phase sample from the liquid product phase; major peaks are named; ISTD = pentadecane; the peaks put together in “phenols” include phenol, cresols, and xylenols.

The first task was to determine which aromatic monomers achieved the highest yields. The GC–MS spectrum (*T*_R_ = 350 °C, *t*_R_ = 20 min; see Figure 6) shows typical degradation products from lignin. Interestingly, we found significantly more catechols (aromatic rings with two attached hydroxy groups) than phenol and other aromatic compounds with one hydroxy group (10 to 20 times higher yields). This does not coincide, for example, with the studies of Belkheiri et al.^61,62^ in which the proportion of phenol in the organic phase is significantly higher. A study with more comparable results is that of Forchheim et al.^42^ The peaks between 12.5 and 15 min of retention time in the chromatogram represent the di- and trimethylcatechols. The problem here is that the underlying NIST database contains only multialkylated resorcinols and hydroquinones, isomers of catechol. However, since only catechol could be found in all samples, the detected compounds are more likely to be alkylated catechols. The mass spectra of the individual peaks clearly show alkyl fragmentation. For the possible dimethyl catechols, the main peaks are *m*/*z* = 123 and *m*/*z* = 138, for the possible trimethyl catechols, they are *m*/*z* = 137 and *m*/*z* = 152 (CH_3_ fragment: *m*/*z* = 15). It is also possible that a small share of ethyl and propyl chains is involved, but for the sake of simplification, only methyl chains were considered. Since it was not possible to analyze these substances individually, a “semi-quantification” was based on the clearly determinable peaks of the catechol and the singly methylated catechols.

**Figure 7:**
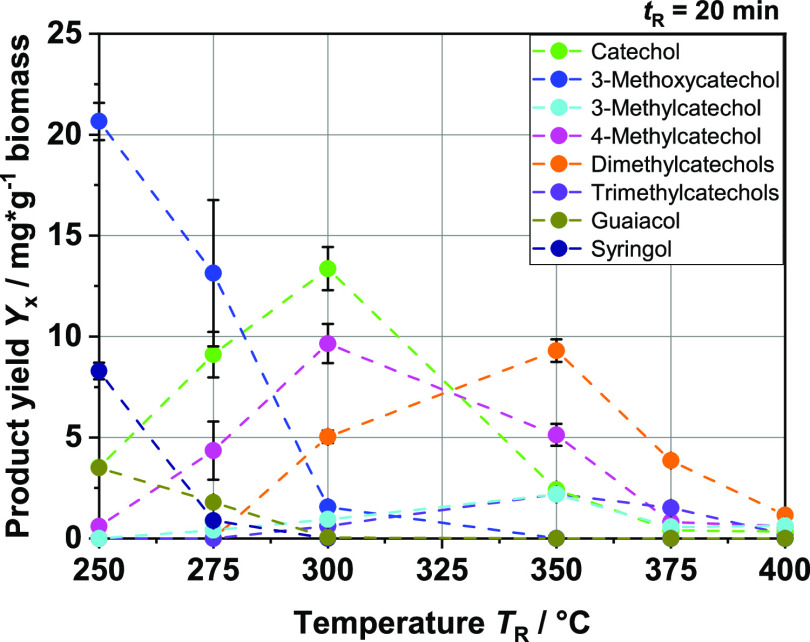
Product yields in mg per g biomass of the main aromatic monomer compounds at different reaction temperatures *T*_R_; feedstock: BL.

Figure 7 shows the calculated yields of the various relevant phenolic monomers obtained in the range *T*_R_ = 250–400 °C at *t*_R_ = 20 min from the batch tests. Phenol itself, as well as cresols and xylenols, hardly plays a role and is not shown in the graph due to the very low measured yields. Basically, the aromatic monomers formed in the reaction can be divided into three sections on the basis of their different functionalities. The first to be generated are phenolic aromatics with at least one hydroxy (–OH) and one methoxy (–OCH_3_) group. These include guaiacol and syringol, which can be derived from the two main building blocks, coniferyl and sinapyl alcohol. Interestingly, comparing the yields shows that the ratio of the two aromatics also matches the ratio of the building blocks in the lignin used. In addition, at lower reaction temperatures, 3-methoxycatechol was quantified at higher concentrations up to a yield of 20 mg/g of biomass. As the reaction temperature *T*_R_ increases, the yields of these three substances decrease rapidly. From *T*_R_ = 350 °C, none of these components could be detected in the extracted organic liquid phase. Afterward, the yields of the various catechols increase sharply. Pure catechol with two hydroxy groups falls into the second category. The catechol yield reaches a maximum at *T*_R_ = 300 °C and then drops steadily at higher temperatures. Although 4-methylcatechol behaves similarly, the decline in yield is slower. Therefore, this molecule, along with the other remaining aromatics shown in the graph, falls into the third range, that of alkylated catechols bearing at least one alkyl group in addition to the two hydroxy groups of the catechol. This group represents the final products of the aromatic monomers obtained from the depolymerization of lignin under hydrothermal conditions. With further increasing reaction temperatures up to *T*_R_ = 400 °C, the yields approach zero. In principle, it can be stated that the production of aromatics is possible from BL directly used as a feedstock in a HTL process without further precipitation of lignin. The lignin molecule is depolymerized as already described in other research papers^31,42^ and forms various, mainly phenolic monomers. In our case, different derivatives of catechols are mainly formed. All catechols together lead to a maximum yield of around 30 mg/g of biomass at 300 °C. Since the yields are generally low, it is necessary to optimize this process for aromatic production. In order to accomplish this, it is first necessary to understand in more detail what happens to lignin under the prevailing conditions.

### Batch HTL Experiments with Model Substances

**Figure 8:**
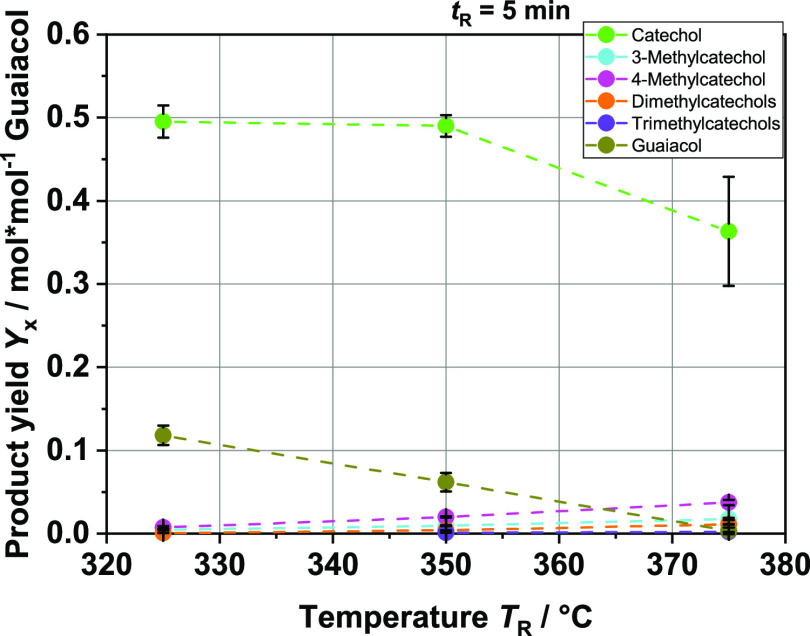
Product yields in mol per mol guaiacol of the main aromatic monomer compounds at different reaction temperatures *T*_R_.

**Figure 9:**
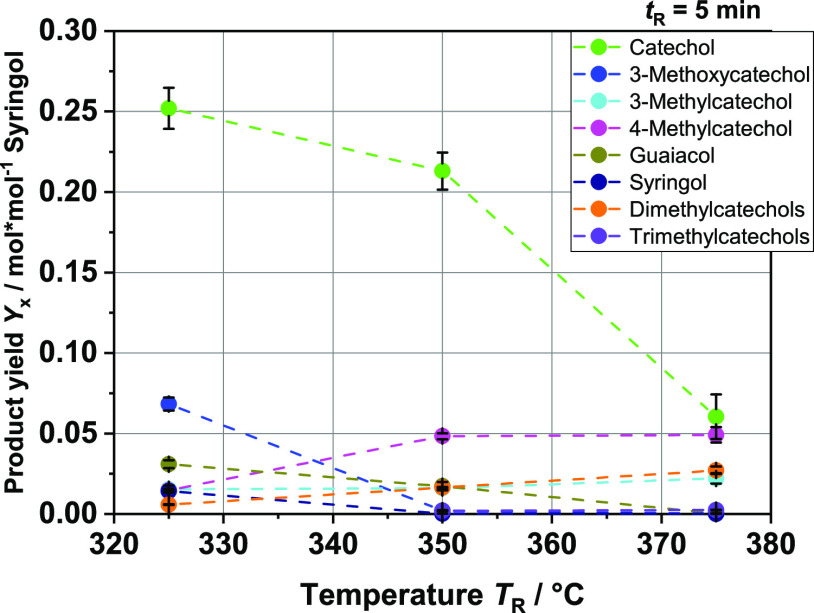
Product yields in mol per mol syringol of the main aromatic monomer compounds at different reaction temperatures *T*_R_.

Since numerous products are formed from lignin that cannot be clearly assigned to one reaction pathway, we decided to simplify the feedstock and work with suitable model substances. We were primarily interested in guaiacol and syringol, which are parts of two basic building blocks of lignin. Figure 8 (guaiacol) and Figure 9 (syringol) show the yields of the aromatics obtained from the HTL of the model substances. If guaiacol is used, then a yield of 50% catechol is possible. Much more interesting, however, is the aspect that in addition to catechol and the unreacted guaiacol, only methylated catechols occur as secondary products. While the yield of catechol decreases slightly at *T*_R_ = 375 °C, their yields increase steadily with increasing reaction temperature. Neither syringol nor 3-methoxycatechol could be detected, strongly suggesting that demethylation of the methoxy group takes place. Methane as a byproduct in the gas phase is quantifiable. Increasing methane concentrations in the complex reaction mixture could subsequently lead to an increased production of methylated catechols. How the methylation of catechols actually proceeds is not clear. Possibly, different radicals, such as radical aromatics or methyl radicals, are formed under the prevailing conditions and can react with each other. Forchheim et al. worked out a simple consecutive reaction pathway via guaiacol under near-critical conditions in^40,42^ and discussed a change in the reaction mechanism from hydrolysis at subcritical temperatures to radical-induced degradation at near-critical and supercritical temperatures. Additionally, the activation energies were close to those for pyrolysis, in which mainly radical reactions take place.

Using syringol instead of guaiacol as a model substance provided further interesting insights. Overall, significantly more compounds were found by GC. While the main product remains catechol, the yield is about half that of guaiacol; 3-methoxycatechol and guaiacol are also form, among others. This allows us to single out two reaction pathways occurring concurrently. On the one hand, the demethylation already mentioned takes place. While guaiacol produces catechol, syringol produces 3-methoxycatechol from this reaction. This is indicated by the absence of 3-methoxycatechol in the experiments with guaiacol. Further experiments with vanillin (based on guaiacol) and syringaldehyde (based on syringol) confirmed this hypothesis. Only experiments with syringaldehyde were shown to produce 3-methoxycatechol. The other parallel reaction is the single demethoxylation of syringol, leading to guaiacol. Methanol is formed as a byproduct, which has been detected in the liquid product. A second demethoxylation reaction to phenol is also conceivable but hardly plays a role due to the small amounts of phenol. According to the results, the first mechanism mentioned, demethylation, seems to proceed somewhat faster since the yield of 3-methoxycatechol is higher. However, these reactions, demethylation and demethoxylation, might proceed in parallel on the same monomer. The significantly higher yield of catechol compared with all other possible molecules is a clear indication of this. In connection with the production of methylcatechols, transalkylation may also play an important role.^63,64^ This is applicable to the pathway of guaiacol as well as to the pathway of syringol. In the work of Zhu et al., for the transalkylation of anisole in the context of a hydrodeoxygenation on acid sites of the catalyst, twice-methylated phenols (xylenol) are also given as products.^65^ Thus, twice or three times methylated catechols based on syringol cannot be excluded. Further studies are needed to clearly define the predominant reaction pathway(s) and evaluate the complex interactions within the reaction network. For example, one approach would be to focus on the two byproducts generated via demethylation (CH_4_) and demethoxylation (CH_3_OH). However, this will not be straightforward with BL, since both methane and methanol can be formed from many other reactions occurring in parallel, e.g., from hemicellulose (compare Figure 12). The results obtained from the experiments with the model substances can be transferred to the HTL experiments with BL and allow us to understand the observed product distribution better.

### Continuous HTL Experiments with BL

**Figure 10:**
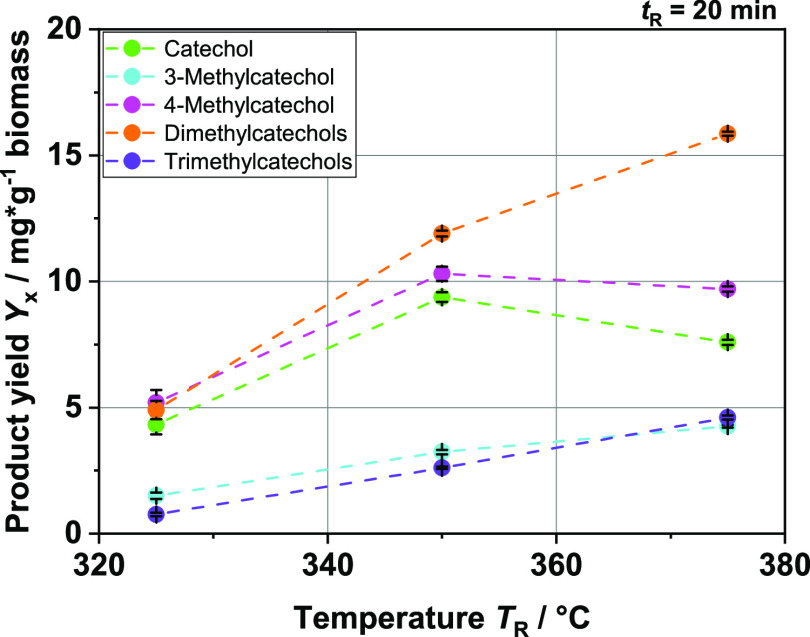
Product yields in mg per g biomass of the different catechol compounds at different reaction temperatures *T*_R_; continuous experiments; feedstock: BL.

Comparative tests were performed in a related continuous plant and confirmed the trends observed in batch tests. We were able to detect the same products in the extracted organic phase, catechol and its derivatives also being the main products (see Figure 10). The yields are slightly higher than those in the batch experiments. At *T*_R_ = 375 °C and a residence time of *t*_R_ = 20 min, well over 30 mg/g of biomass catechols could be observed. Interestingly, the dimethyl catechols play a much larger role. Moreover, the yields of all methylated catechols remained stable or increased in the temperature range studied. In contrast, at *T*_R_ = 375 °C in the batch experiment, the yields had already decreased significantly. However, a direct comparison at the same temperature and residence time is difficult since the heat transfer to the feedstock in the reactor is much better in the continuous plant than in the reactors operated batch-wise. During the batch experiments, the heat must first pass through the reactor wall to the center of the fluid, whereas in the continuous reactor, an additional heat carrier stream of hot water presumably heats small droplets of feedstock from all sides. The same applies to subsequent cooling. Therefore, it can be assumed that reactions in the continuous system can take place in a much more segregated manner with a far more limited number of side reactions than in the batch system. Thus, for example, repolymerization with solids may be limited, allowing the preferential formation of alkylated catechols. The methylation itself is not limited in the continuous process since it is a direct consecutive reaction and methyl radicals may not be diluted as much as in as in a batch system. In fact, it is even more pronounced than in the batch experiment results. Therefore, it is likely that a reaction scheme setup on the basis of the batch experiments also applies to the continuous process since we could observe the same ongoing reactions with both experimental designs.

### Discussion of Carbon Mass Balance

**Figure 11:**
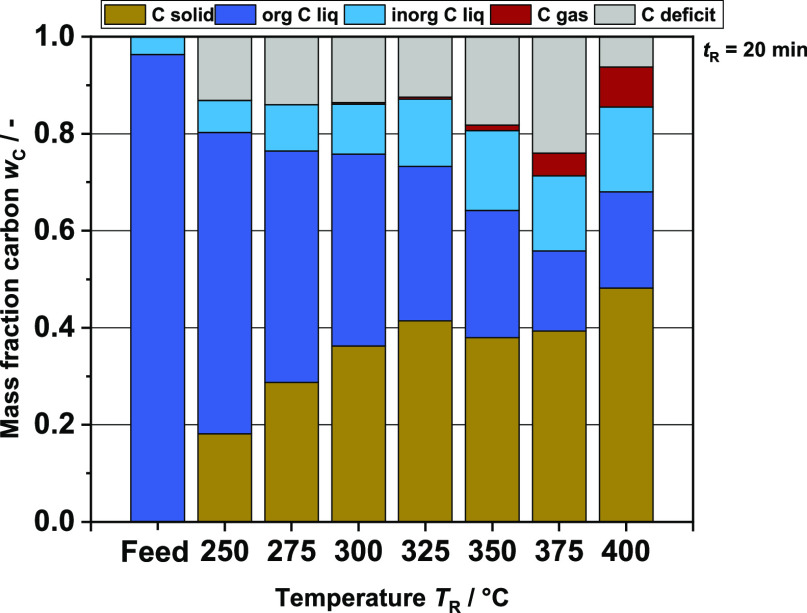
Carbon mass fractions at different reaction temperatures *T*_R_ and before processing (feed); brown: C mass fraction solid; green: organic C mass fraction liquid phase; blue: inorganic C mass fraction liquid phase; red: C mass fraction gas phase (calculated from the detected gas compounds); and gray: deficit of carbon.

**Figure 12:**
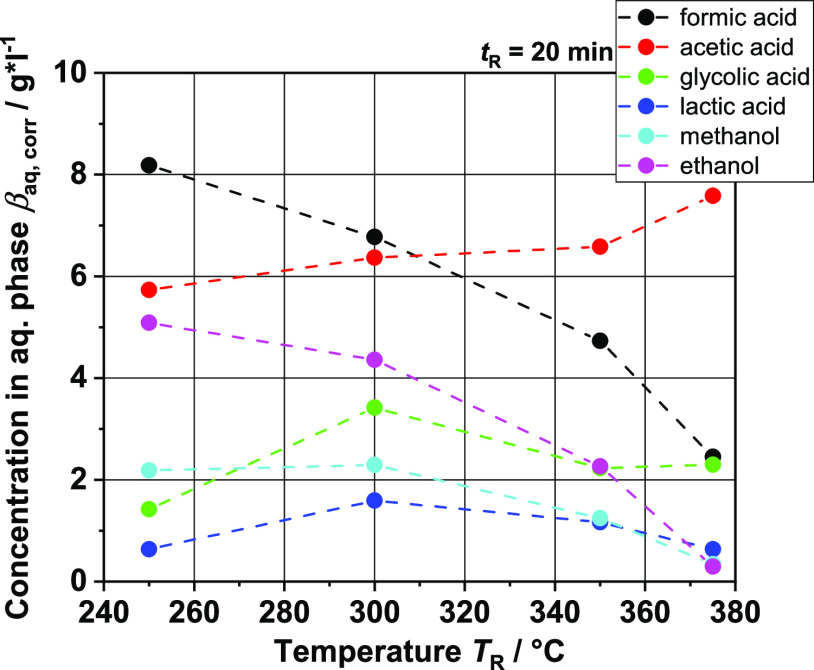
HPLC analysis of aqueous phase after extraction of the organic phase; ethyl acetate concentration (extract solvent) was subtracted to calculate corrected mass concentrations.

With the results shown so far, a reaction network can be developed on the monomer side, specifically for the case studied here in this work. This fits well with other studies in the field. However, the low yields of monomeric compounds indicate that it is not sufficient to focus on only aromatic monomers. A first assumption would be that at a high reaction temperature *T*_R_, the regime of hydrothermal gasification is achieved, and therefore many organic compounds are in the gas phase.^66^ However, considering the carbon mass balance for the batch experiments (Figure 11), it quickly becomes clear that this is not the case. The decrease in organic carbon in the liquid phase observed with increasing reaction temperature *T*_R_ (dark blue) is not reflected in an increase in the carbon share in the gas phase (red). In comparison, the carbon content in the solid product shows a significantly larger increase. At *T*_R_ = 400 °C, this accounts for about half of the total carbon. At first glance, the observed phenomenon does not match the actual behavior of hydrothermal processes as hydrothermal carbonization is instead located in the lower reaction temperature range. However, monomers and oligomers can repolymerize rapidly due to the high density of functional groups^44,67^ present in the material. Branched hydrocarbons and aromatics in particular seem to contribute to the production of high molecular weight substances such as coke, coal, or tar.^68^ It is known from catalytic cracking, for example, that aromatics can quickly coke and thus deactivate the catalyst.^69^ Ultimately, repolymerization side reaction effects lead to the fact that hardly any aromatic monomers are found in the liquid product phase at high temperatures. About 50–60 wt % of the organic carbon in the liquid product phase (dark blue column section in the C balance) is detected via GC-FID and HPLC (see Figure 12) monomer analysis. This strongly suggests that almost half of the carbon present in liquid organic products after extraction is bound in aromatic oligomers or even larger molecules. Thus, in addition to the solid, this part must also be taken into account, since the organic liquid phase produced is the desired product of the process. The carbon deficit shown in the graph is most likely due to gaseous or volatile compounds that could not be detected by gas analysis. Another reason for the loss of carbon is residues in the reactor after opening.

### Comparison between Softwood and Hardwood BL as HTL Feedstock

**Figure 13:**
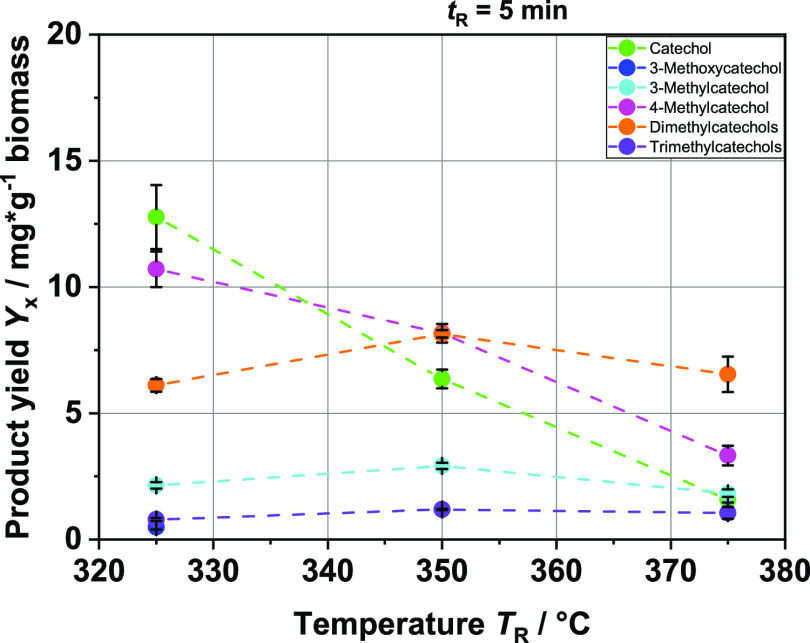
Product yields in mg per g biomass of the different catechol compounds at different reaction temperatures *T*_R_; batch experiments; and feedstock: hardwood BL.

**Figure 14:**
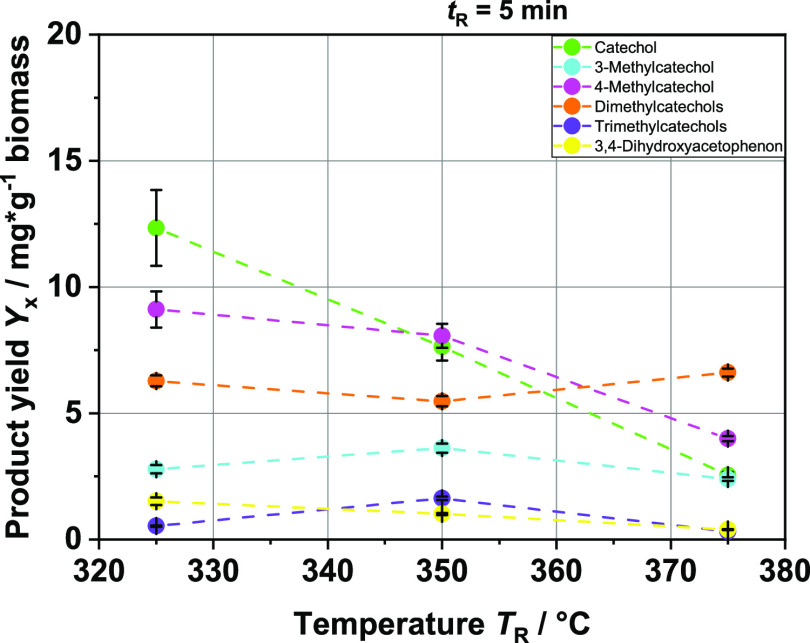
Product yields in mg per g biomass of the different catechol compounds at different reaction temperatures *T*_R_; batch experiments; and feedstock: softwood BL.

**Figure 15:**
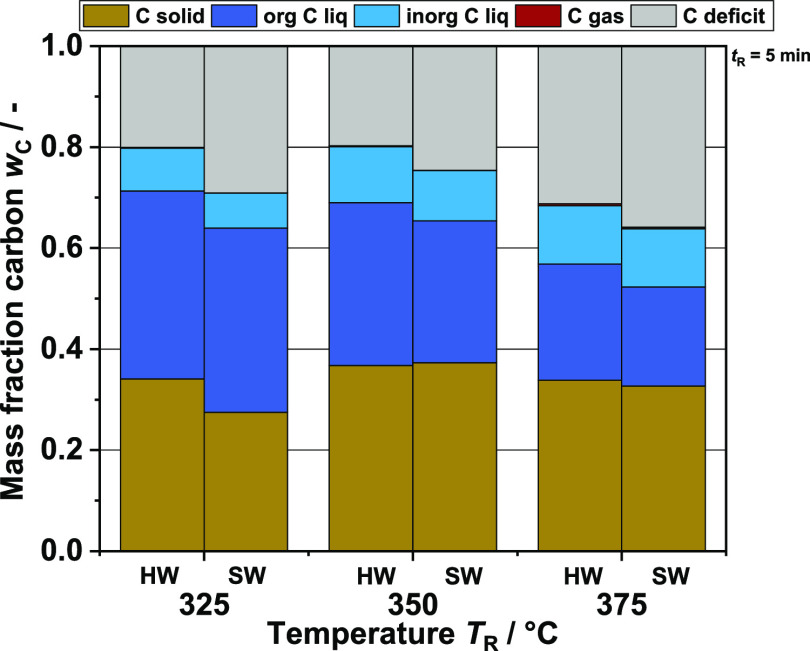
Carbon content in different product phases of batch experiments with hardwood-based (HW) BL and softwood-based (SW) BL at three different reaction temperatures.

In addition to batch experiments with BL based on hardwood, we carried out experiments with BL based on softwood. In this way, we want to show that a reaction scheme based on the results shown so far is applicable for different types of BL as a feedstock. Figures 13 and 14 show the yields of the aromatic monomers. In fact, the products hardly differ, either qualitatively or quantitatively. The only interesting differences are those that we also observed in the HTL of the model substances. The 3-methoxycatechol, which we were able to detect in the experiments with syringol, is only present in the products of the HTL with the Hardwood BL. This makes sense since hardwood, as already mentioned, is composed of a significantly larger proportion of sinapyl alcohol, the molecule from which syringol is formed (around 70:30 S to G ratio). Softwood, on the other hand, consists almost exclusively of the building block coniferyl alcohol, the derivative of guaiacol (around 90–95% G). Thus, the reaction pathway is only via the guaiacol, and 3-methoxycatechol is not formed. Since the carbon mass balances (see Figure 15) are also very close to each other, it can be concluded that the wood type does not have a major effect on the HTL and its aromatic monomers as products. Instead, the salts probably have a much greater effect. It appears that in the softwood, the syringol is skipped in the sequence of reactions, but this ultimately has no visible effect on the yields of the different catechols. Therefore, it is probably possible to apply the reaction scheme to different BLs as long as the salt concentration does not deviate too much. The high loss of carbon shown in the mass balances is most likely a result of the issues mentioned above in the discussion about Figure 11. The statement about the negligible difference in the yields of the produced aromatics should not be affected by the carbon loss, since the experiments with softwood and hardwood BL share the same issue.

### NMR Analysis of Liquid and Solid Product Phase from HTL

**Figure 16:**
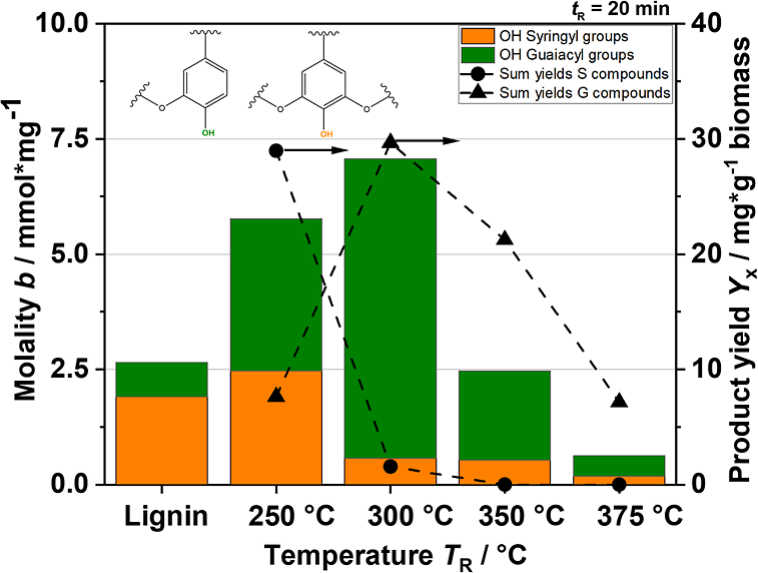
Molality of OH groups in syringyl groups and guaiacyl groups (see chemical structures in the figure) and the product yields of summed up S compounds and G compounds at different reaction temperatures.

In order to gather more information about the oligomers in the organic product, we searched for suitable parameters that could describe the evolution of these larger molecular structures. One possibility is the quantification of the hydroxy groups (–OH) present in lignin and its depolymerization products. Using a specific phospholysis derivatization technique followed by ^31^P NMR analysis as described in the Materials and Methods Section, it is possible to assign hydroxy groups quite precisely and, moreover, to quantify them to individual molecular groups depending on the observed chemical shifts. The high efficiency of the derivatization technique allows us to treat the extracted organic phase from the liquid product and assess all the hydroxy groups present in the complex reaction mixture. In Figure 16, the molality of the hydroxy groups is shown on the left *Y*-axis. We focused on guaiacyl-OH and syringyl-OH because they compare well with our quantified monomers. It is difficult to distinguish them from the catechols because the NMR signals partially overlap. Therefore, we assumed that the 3-methoxycatechol signal is located close to the syringyl group and that the remaining catechols could be assigned to the guaiacyl group.^70,71^ Due to the low mass of extracted biocrude, only four sample analyses and the feedstock analysis (extracted lignin) could be meaningfully evaluated. Nevertheless, the results of the ^31^P NMR analysis are very interesting and fit well with the previous results. As expected, the lignin consists of a much larger fraction of syringyl groups since we are working with hardwood lignin.^72^ As soon as the depolymerization of the lignin molecule starts, the molality of the hydroxy groups increases. This is due to the fact that β-O-4 bonds are preferably broken in the lignin structure, which presumably results in the formation of further hydroxy groups. From *T*_R_ = 300 °C, hardly any syringyl-OH is present in the mixture, while significantly more guaiacyl-OH is produced. For comparison, the summed yields of the individual aromatics from GC-FID are plotted on the right *Y*-axis in the same Figure 16. The calculated amounts are related to the measured ^31^P NMR regions, meaning that 3-methoxycatechol is considered together with syringol as S-components and, similarly, the remaining catechols together with guaiacol as G-components. Only at *T*_R_ = 250 °C is the ratio of the yields of the S-components and the G-components significantly higher compared to the ratio of the molality of syringyl-OH and the guaiacyl-OH. This is possibly due to a “dual” role of 3-methoxycatechol: it is quite possible that the second OH-group present in the molecule produces a signal in the guaiacyl group range and its share is therefore in the guaiacyl-OH molality at the same time. Another reason may be oligomers, which are soluble enough to be detected using NMR but not volatile enough to be evaluated via GC. Nevertheless, the overall results are in good agreement, validating the chosen derivatization technique. Hence, it can be concluded that the reactions involving the hydroxy groups of the oligomers behave similarly. This again means that with increasing temperature, the hydroxy group is the predominant functional group on all aromatic structures within the biocrude. The behavior of the oligomeric organic structures in the liquid product phase is therefore clarified, and the same reaction scheme can be used for the monomers.

**Figure 17:**
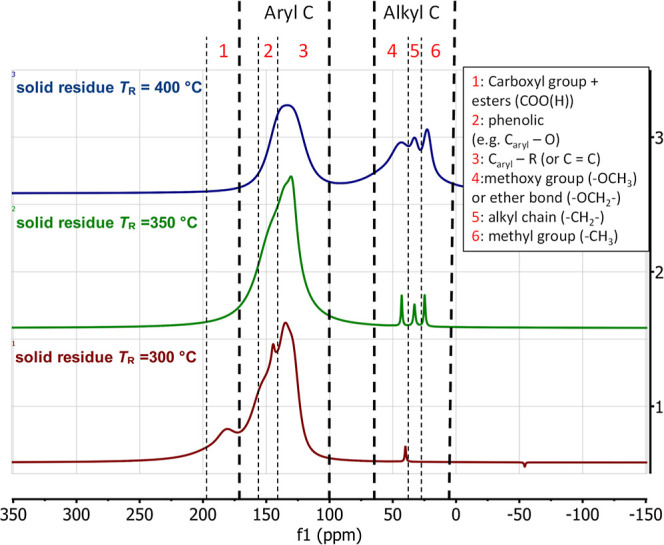
^13^C solid-state NMR spectra for the solid phase at three different reaction temperatures.

Looking back at the carbon mass balance, it is noticeable that a large proportion of carbon ultimately ends up in the solid product. This is not desired but could not have been avoided with the parameters investigated. In order to understand what ultimately ends up in the solid, we analyzed three solid samples from three different batch tests (*T*_R_ = 300, 350, and 400 °C) using ^13^C solid-state NMR. The three NMR spectra produced can be seen in Figure 17. Two main signal regions are clearly visible. The region in the low field (left in the spectra, 120–160 ppm) shows ^13^C carbons present in aromatic compounds (aryl-C), and the region in the higher field (right-hand region group 25–50 ppm) shows carbon in alkyl compounds (alkyl-C). A region worthy of note can be found in the lower field, typical of carboxyl groups (carboxylic acids and derivatives like esters or amides: ranging from 170 to approximately 185 ppm) at *T*_R_ = 300 °C. However, a signal at 180 ppm disappears at higher temperatures. The aromatic peak itself probably consists of an aryl-C–O-peak, which, however, is only visible clearly at *T*_R_ = 300 °C, in addition to the pure aryl C-peak. The aryl-C–O bond fits to the phenolic structure of the lignin. At higher temperatures, the signal of this bond could be overshadowed by the aryl-C peak. An indication is the width of the peak, which looks like two signals merged together. The peak, which shows the aromatic bonds, could also be generated by C=C bonds. Since we were not able to observe carbon double bonds in any other analysis, we assume that the peak describes the aromatic carbon. In general, this signal indicates that the solid is preferably built up from aromatic building blocks. This is a consequence of using lignin as feedstock and its products. Likewise, it supports the assumption that the aromatics are a driver of repolymerization and preferentially remain in the solid product. Moreover, the ^13^C solid-state NMR spectra fit very well with the observed yields of the monomers. We observed that the yields of all aromatic monomers decreased with an increasing reaction temperature. We also saw that methoxy and hydroxy groups were preferentially present at lower reaction temperatures, and methyl groups were more prominent as the reaction temperature increased. The same pattern can be found in the NMR spectra for the solid product. At *T*_R_ = 300 °C, the aromatic peak is clearly dominant. At a high reaction temperature of *T*_R_ = 400 °C, the ratio of the aromatic peak to the alkyl peak is almost balanced. This strongly suggests that the monomers formed without a methyl group repolymerize first, and only later do the aromatics with an alkyl radical formed at higher reaction temperatures also appear. Another hypothesis is that reaction mechanisms similar to those for the monomers also apply to the solid, as already shown for the oligomers.

### Investigation of the Molecular Mass of the Biocrudes via SEC Analysis

**Figure 18:**
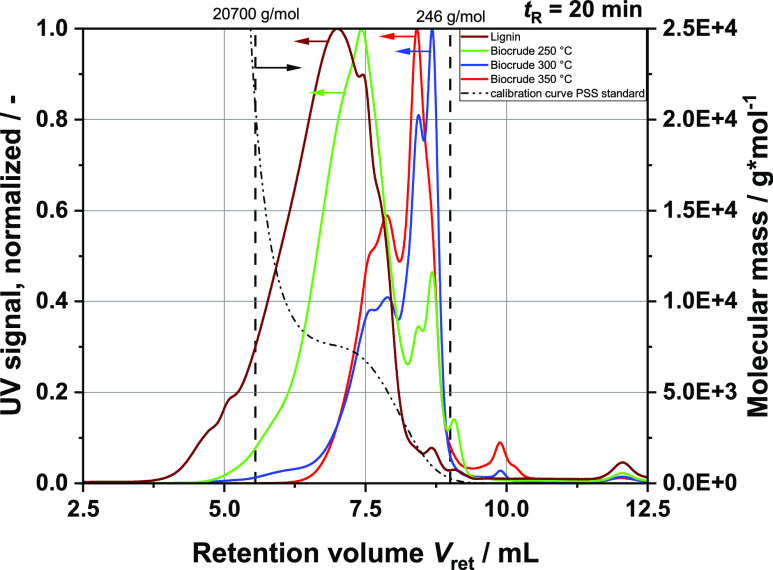
SEC analysis of different extracted biocrudes and lignin; left *Y*-axis: UV signal over retention time; right *Y*-axis: molecular mass over retention time; and calibration curve (dashed-dotted line) with the calibration minimum and maximum (vertical dashed lines at 20700 and 246 g mol^–1^).

**Figure 19:**
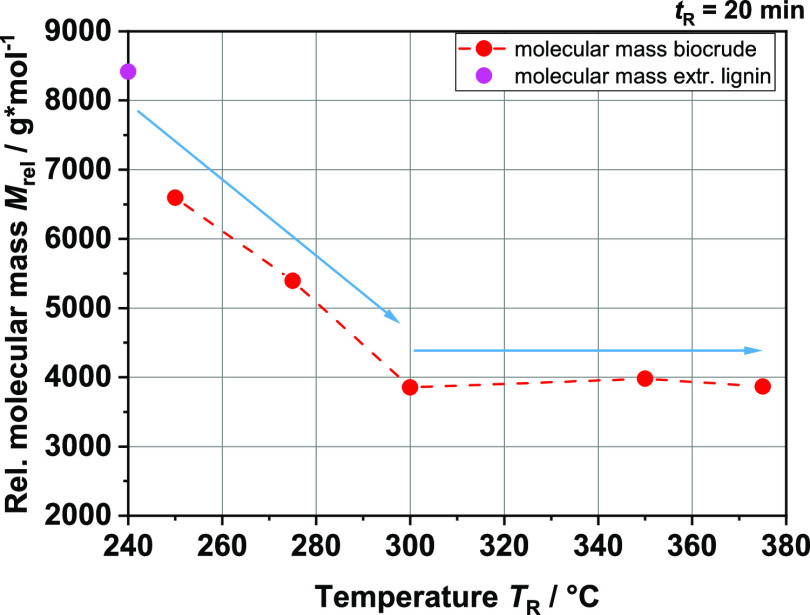
Relative molecular mass of extracted biocrudes at different reaction temperatures (red) and the molecular mass of extracted lignin from BL (pink).

Another option to describe the depolymerization of lignin is to assess the molar mass of the molecules present in the extracted biocrude. The results of the SEC analysis are summarized in Figure 18, allowing for easy determination of the relative molecular mass *M*_rel_. The UV signals of three biocrudes from batch experiments performed with different reaction temperatures *T*_R_ are plotted (together with the related calibration curve) versus the retention volume *V*_ret_, the volume of eluent passed through the column. The column used was calibrated in the range of 246 to 20,700 g mol^–1^. The recorded eluent volumes are within the two dashed lines (minimum and maximum). This region delivers the most conclusive data. Only the lignin displays signals below the minimal limit, somehow impeding interpretation. It can be clearly observed how depolymerization progresses with increasing temperatures. The maximum peaks clearly shift to the right toward smaller molecular weights. New prominent peaks form at the three investigated reaction temperatures around 1000 and 1500 g mol^–1^. This would correspond to oligomers displaying 7 to 10 syringyl groups (see Figure 16). The beginning of repolymerization can also be observed. For instance, the peak around 1000 g mol^–1^ is no longer present in the product obtained at *T*_R_ = 350 °C. Instead, the 1500 and 4000 g mol^–1^ peaks are much more pronounced. Monomers cannot theoretically be observed with the setup used, with the calibration range taken into consideration. However, the functional groups can interact with the column and lead to retention time shifts. Therefore, the measured molecular mass will be labeled as the relative molecular mass for the remainder of this paper. Finding a suitable setup for SEC analysis of lignin is difficult due to the dual polar and nonpolar character of the feed, without even first modifying the molecules, e.g., with acetylation. However, the idea was to gain direct information about the molecule sizes. Nevertheless, some trends can be detected by investigating depolymerization and repolymerization behavior. Figure 19 shows the weight-averaged relative molecular weight of various biocrudes. Up to *T*_R_ = 350 °C, a significant decrease can be seen. Thereafter, interestingly, *M*_rel_ remains constant with an increasing reaction temperature *T*_R_. It can be assumed that some kind of equilibrium is set between depolymerization and repolymerization. We assume that although the lignin molecule is cleaved into smaller molecules, the high temperatures used rapidly lead to partial repolymerization. One reason for the faster repolymerization reactions could be radical reactions, for example, which are known to occur more frequently at higher temperatures.^40^

### Development of a Simplified Reaction Scheme

**Figure 20:**
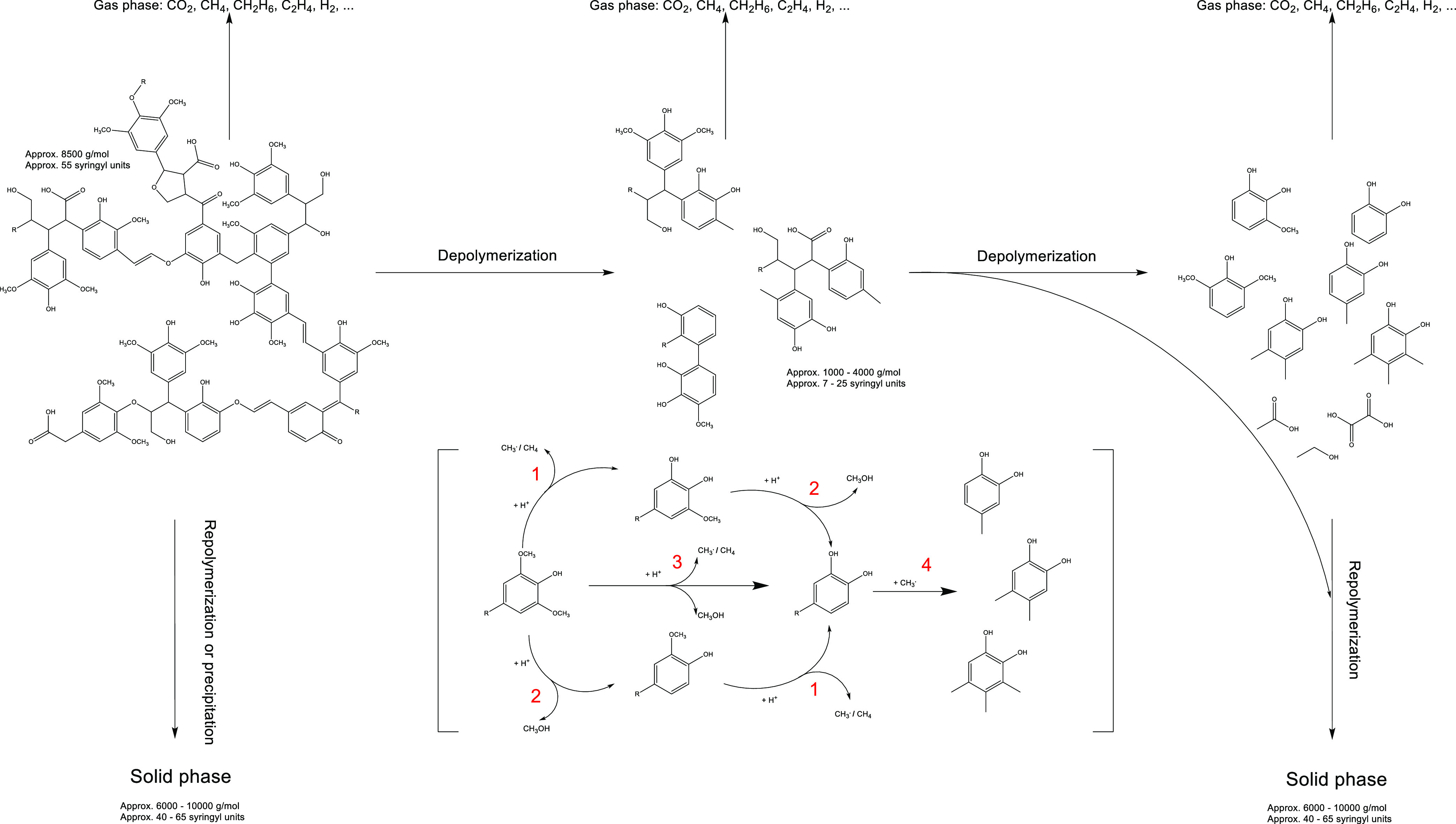
Reaction scheme of lignin depolymerization during HTL of BL based on the results of the experimental work, reaction mechanism in parentheses applies to all of the three groups (lignin, oligomers, and monomers); lignin structure on the right reproduced with permission from ref (18). Copyright 2013 Elsevier.

Based on the results gathered from the comprehensive analytics, a simplified reaction scheme can be tentatively proposed (Figure 20). Compared to the reaction scheme proposed by Forchheim et al.,^42^ the monomer products are somewhat different. In our work, the depolymerization of lignin by HTL under the influence of the cooking chemicals present in BL produces catechols as the main products. The reaction network includes three different reaction pathways, starting from syringol to catechol. They are shown together with the subsequent alkylation in parentheses. These reactions can happen on monomers, oligomers, and the lignin itself. The main reactions are demethylation (1) and demethoxylation (2). The byproducts of the first of these reactions are methane and methyl radicals, whereas the latter leads to the formation of methanol. Detecting 3-methoxycatechol in the product produced at *T*_R_ = 250 °C or *T*_R_ = 275 °C, it can be assumed that demethylation proceeds preferentially in this temperature range. At higher temperatures, however, the difference hardly seems to exist since the yields of catechol and its derivatives clearly exceed those of the remaining oxygenated aromatics. Thus, in the range of *T*_R_ = 300 °C or higher, it can be assumed that both reactions can proceed simultaneously (3). In contrast, two similar reactions with the methoxy groups of the syringol are not the preferred reaction pathway. As confirmation, 1,2,3-trihydroxybenzene (pyrogallol), the product of a double demethylation of syringol, could be detected in the GC–MS spectrum, although the amount was too small for quantification. Similarly, phenol is produced, resulting from a double demethoxylation of the syringol. Phenol could be quantified, but the yields were much lower than those of catechols. Therefore, it appears that two different reactions starting from syringol are preferred.

After demethylation and demethoxylation, alkylation of catechol, mainly methylation (4), occurs more with increasing reaction temperature *T*_R_. As already discussed, radical reactions with methyl radicals are probably responsible for this. The methyl radicals may be formed during demethylation, for example. Another possibility is transalkylation, in which a methylcatechol and a catechol molecule can be formed from a catechol and a guaiacol molecule, for example. The experimental data generated does not allow us to determine the extent to which the transalkylation reaction or the abovementioned radical reactions are ultimately decisive for the increase in methylated catechols. Using ^31^P NMR analysis, we were able to show that the reactions of the monomers also proceed in a manner similar to that of the functional groups of the oligomers. It can be assumed that methylation also takes place within the oligomers. For the sake of simplification, it makes sense to apply the reaction pathways proposed for the monomers to the structurally related oligomers as well.

In addition to aromatics as the main components, many alcohols and acids are also formed on the monomer side. The formation of such acidic compounds can be explained not only by the cleavage of a wide variety of side groups, which occurs, for instance, in the demethoxylation mentioned above, but also from other components of the BL, such as hemicellulose. The organic acids and alcohols produced predominantly end up in the aqueous phase of the liquid product after extraction. In a technical approach, it would make sense to look for ways to utilize the aqueous phase. There is a lot of ongoing research into aqueous phase reforming, for example.^73^ Another strategy is the recovery of phenolic compounds that did not transfer to the organic phase during extraction. A new methodology using hydrophobic eutectic solvents based on different mixtures of terpenes (menthol and thymol) and organic acids (octanoic acid, decanoic acid, and dodecanoic acid) was studied by Pola et al.^74^

In the overall picture, the depolymerization of lignin under the conditions given in the research work results in three main phases. The gas phase is negligible regarding the carbon content but can be crucial for alkylation via the methane or methyl radicals. Typical gas phase compounds are CO_2_, H_2_, and different small hydrocarbons. The two main product phases are the liquid and solid phases, which are closely linked. Presumably, small and larger oligomers or even monomers are separated from the lignin. Specific bonds break during this step, e.g., the remaining β-O-4 bonds. The same happens with the oligomeric structures in the following step, which ultimately produces more monomer compounds. It is possible that a certain portion of the lignin ends up directly in the solid due to precipitation and does not actively participate further in the depolymerization. It can likewise be assumed that as the reaction temperature *T*_R_ rises, the proportion of oligomers and monomers that react via repolymerization to form larger molecules that ultimately end up as solids increases sharply. Conversely, it is rather improbable that a material flow from the solid will return to the liquid phase. The different relative molecular weights can be used to show how the size ratios of the individual groups compare with each other. In the end, the solid does not differ much from the lignin itself, at about 8500 g mol^–1^. In the case of the oligomers, there are various intermediates, as shown by the SEC analysis. The monomers have relative molecular weights in the range of 90–200 g mol^–1^. A potential explanation for the intermediates could be the different activation energies of the bonds within the molecule.

## Conclusions

In the present study, we were able to show that BL can be used directly in a HTL process for the production of aromatics. Derivatives of catechol were identified as the main products of depolymerization of the lignin dissolved in the BL, diverging from the results reported by numerous research groups that mentioned phenol as one of the main components. However, the yields produced remain low, in relation to the biomass used. Consequently, fruitful development of this process requires significant enhancement of the selectivity toward catechol and, on the other hand, better valorization of the main byproducts generated by the BL treatment. An important step in further developing the HTL is to better understand how depolymerization occurs. By calculating the yields of various monomers, three main reactions have become apparent. Starting from the lignin structure, it is demethylation and demethoxylation that lead to catechol. The catechol is then alkylated in the next step, probably by radical reactions or transalkylation catalyzed by the various salts present in the reaction mixtures. High temperatures (above *T*_R_ = 350 °C) seem to accelerate repolymerization reactions, leading to a significant yield reduction. Regarding the lignin depolymerization, the behavior of the oligomers must also be thoroughly investigated. By carrying out NMR analyses, we were able to prove that the oligomers and their functional groups also participate in demethylation and demethoxylation reactions, which take place in a way similar to that observed with monomers. This greatly facilitates the establishment of the reaction scheme since the postulated reaction pathways can be adopted for the monomers in a lumped reaction scheme. The possibility of describing the depolymerization sufficiently on the basis of functional groups, such as the hydroxy groups, without a loss of relevant information is a great step forward. The first positive trends observed so far now have to be investigated in greater depth, for instance, with kinetic models and specific series of experiments. In addition, we used SEC analysis to determine a further parameter for describing the process. In doing so, we were able to illustrate once again how important it is to suppress repolymerization, as otherwise, no further progress can be seen in reducing the size of the molecules. This obviously leads to a high loss of valuable carbon in the solid phase. Lastly, we were able to show that in a direct HTL of BL, the cooking chemicals have a much greater influence than the wood species dissolved in the BL. The results between softwood and hardwood hardly differ. This clearly indicates that the reaction scheme can be applied not only to one specific BL but also to other liquors as long as the composition of the cooking chemicals is somewhat similar.

## Supporting Information Available

Weighed-in masses of salts used for preparation of 100 mL of salt solution; flow scheme of the micro autoclave station to open and close the autoclaves; volumes of BL used at specific reaction temperatures *T*_R_; explanation of the calculation of the carbon mass in the gas phase; pressure correlation in micro autoclave station; data for calculation of the used coefficient in eq 1 to calculate *p*_corr_; equations for calculation of the carbon mass in the gas phase; gas compounds which are detectable with the used GC setup (TCD and FID); experimentally determined distribution coefficients for different monocyclic phenolic compounds when using the described extraction procedure; and detailed information about the ^13^C NMR analysis (DOCX)

## Abbreviations

*T*_R_: reaction temperature
*t*_R_: holding time (batch)/residence time (cont.)
BTX: benzene, toluene, xylene
MtA: methanol to aromatics
BL2F: black liquor to fuels
HTL: hydrothermal liquefaction
NMR: nuclear magnetic resonance
HSQC: heteronuclear single quantum correlation
CP/MAS: cross-polarization magic angle spinning
SEC: size exclusion chromatography
BL: black liquor
GC: gas chromatography
FID: flame ionization detector
TCD: temperature conductivity detector
ISTD: internal standard
*Y*_*x*_: product yield of compound *x*
HPLC: high-pressure liquid chromatography
MS: mass spectroscopy
S: syringyl
G: guaiacyl
*w*_*x*_: mass fraction of species *x*
*M*_rel_: relative molecular mass

## References

[ref1] Straits Research. Benzene-Toluene-Xylene (BTX) Market Size, Share & Trends, Forecasts, 2021–2030. https://straitsresearch.com/report/benzene-toluene-xylene-market (accessed Jan 9, 2024).

[ref2] Levi P. G.; Cullen J. M. Mapping Global Flows of Chemicals: From Fossil Fuel Feedstocks to Chemical Products. Environ. Sci. Technol. 2018, 52 (4), 1725–1734. 10.1021/acs.est.7b04573. 29363951

[ref3] International Energy Agency (IEA). CO2 Emissions in 2022, Paris. https://www.iea.org/reports/co2-emissions-in-2022 (accessed Feb 21, 2024).

[ref4] Schaefer B. Natural Products in the Chemical Industry;; Springer Berlin Heidelberg, 2014.

[ref5] Clark J.; Deswarte F. The Biorefinery Concept. In Introduction to Chemicals from Biomass, 2nd ed.; Clark J. H., Deswarte F. E. I., Eds.; Wiley Series in Renewable Resources; Wiley, 2015; pp 1–29.

[ref6] Ferreira A. F. Biorefinery Concept. In Bio-refineries: Targeting Energy, High Value Products and Waste Valorisation; Rabaçal M., Ferreira A. F., Silva C. A. M., Costa M., Eds.; Lecture Notes in Energy; Springer, 2017; Vol. 57, pp 1–20.

[ref7] Bio-refineries: Targeting Energy, High Value Products and Waste Valorisation; Rabaçal M., Ferreira A. F., Silva C. A. M., Costa M., Eds.; Lecture Notes in Energy; Springer, 2017; Vol. 57.

[ref8] Ragauskas A. J.; Williams C. K.; Davison B. H.; Britovsek G.; Cairney J.; Eckert C. A.; Frederick W. J.; Hallett J. P.; Leak D. J.; Liotta C. L.; et al. The path forward for biofuels and biomaterials. Science 2006, 311 (5760), 484–489. 10.1126/science.1114736. 16439654

[ref9] Cherubini F. The biorefinery concept: Using biomass instead of oil for producing energy and chemicals. Energy Convers. Manage. 2010, 51 (7), 1412–1421. 10.1016/j.enconman.2010.01.015.

[ref10] Ragauskas A. J.; Beckham G. T.; Biddy M. J.; Chandra R.; Chen F.; Davis M. F.; Davison B. H.; Dixon R. A.; Gilna P.; Keller M.; et al. Lignin valorization: improving lignin processing in the biorefinery. Science 2014, 344 (6185), 1246843 10.1126/science.1246843. 24833396

[ref11] del Amo-Mateos E.; López-Linares J. C.; García-Cubero M. T.; Lucas S.; Coca M. Green biorefinery for sugar beet pulp valorisation: Microwave hydrothermal processing for pectooligosaccharides recovery and biobutanol production. Ind. Crops Prod. 2022, 184, 115060 10.1016/j.indcrop.2022.115060.

[ref12] Kumar B.; Bhardwaj N.; Agrawal K.; Chaturvedi V.; Verma P. Current perspective on pretreatment technologies using lignocellulosic biomass: An emerging biorefinery concept. Fuel Process. Technol. 2020, 199, 106244 10.1016/j.fuproc.2019.106244.

[ref13] Conte M.; Lopez-Sanchez J. A.; He Q.; Morgan D. J.; Ryabenkova Y.; Bartley J. K.; Carley A. F.; Taylor S. H.; Kiely C. J.; Khalid K.; Hutchings G. J. Modified zeolite ZSM-5 for the methanol to aromatics reaction. Catal. Sci. Technol. 2012, 2 (1), 105–112. 10.1039/C1CY00299F.

[ref14] Gautam P.; Neha; Upadhyay S. N.; Dubey S. K. Bio-methanol as a renewable fuel from waste biomass: Current trends and future perspective. Fuel 2020, 273, 117783 10.1016/j.fuel.2020.117783.

[ref15] Lourenço A.; Pereira H. Compositional Variability of Lignin in Biomass. In Lignin-Trends and Applications; Poletto M., Ed.; IntechOpen, 2018.

[ref16] Bresinsky A.; Körner C.; Kadereit J. W.; Neuhaus G.; Sonnewald U. Lehrbuch der Botanik, 36. Aufl.; Spektrum Akad. Verl., 2008.

[ref17] Tolbert A.; Akinosho H.; Khunsupat R.; Naskar A. K.; Ragauskas A. J. Characterization and analysis of the molecular weight of lignin for biorefining studies. Biofuels, Bioprod. Biorefin. 2014, 8 (6), 836–856. 10.1002/bbb.1500.

[ref18] Lange H.; Decina S.; Crestini C. Oxidative upgrade of lignin - Recent routes reviewed. Eur. Polym. J. 2013, 49 (6), 1151–1173. 10.1016/j.eurpolymj.2013.03.002.

[ref19] José Borges Gomes F.; de Souza R. E.; Brito E. O.; Costa Lelis R. C. A review on lignin sources and uses. J. Appl. Biotechnol. Bioeng. 2020, 7, 100–105. 10.15406/jabb.2020.07.00222.

[ref20] Schutyser W.; Renders T.; van den Bosch S.; Koelewijn S.-F.; Beckham G. T.; Sels B. F. Chemicals from lignin: an interplay of lignocellulose fractionation, depolymerisation, and upgrading. Chem. Soc. Rev. 2018, 47 (3), 852–908. 10.1039/C7CS00566K. 29318245

[ref21] Tricker A. W.; Stellato M. J.; Kwok T. T.; Kruyer N. S.; Wang Z.; Nair S.; Thomas V. M.; Realff M. J.; Bommarius A. S.; Sievers C. Similarities in Recalcitrant Structures of Industrial Non-Kraft and Kraft Lignin. ChemSusChem 2020, 13 (17), 4624–4632. 10.1002/cssc.202001219. 32539201

[ref22] Metsä Group Metsä Group. Sustainability Report 2018, https://www.metsagroup.com/news-and-publications/news/2019/metsa-groups-2018-annual-review-sustainability-report-and-brochure-published/ (accessed Feb 21, 2024).

[ref23] Alén R. Pulp Mills and Wood-Based Biorefineries. Industrial Biorefineries & White Biotechnology; Elsevier, 2015; pp 91–126.

[ref24] Fache M.; Boutevin B.; Caillol S. Vanillin Production from Lignin and Its Use as a Renewable Chemical. ACS Sustain. Chem. Eng. 2016, 4 (1), 35–46. 10.1021/acssuschemeng.5b01344.

[ref25] Roy R.; Rahman M. S.; Amit T. A.; Jadhav B. Recent Advances in Lignin Depolymerization Techniques: A Comparative Overview of Traditional and Greener Approaches. Biomass 2022, 2 (3), 130–154. 10.3390/biomass2030009.

[ref26] Weng C.; Peng X.; Han Y. Depolymerization and conversion of lignin to value-added bioproducts by microbial and enzymatic catalysis. Biotechnol. Biofuels 2021, 14 (1), 84 10.1186/s13068-021-01934-w. 33812391 PMC8019502

[ref27] Jahn A.; Hoffmann A.; Blaesing L.; Kunde F.; Bertau M.; Bremer M.; Fischer S. Lignin from Annual Plants as Raw Material Source for Flavors and Basic Chemicals. Chem. Ing. Tech. 2020, 92 (11), 1733–1740. 10.1002/cite.202000097.

[ref28] Patwardhan P. R.; Brown R. C.; Shanks B. H. Understanding the fast pyrolysis of lignin. ChemSusChem 2011, 4 (11), 1629–1636. 10.1002/cssc.201100133. 21948630

[ref29] Kibet J.; Khachatryan L.; Dellinger B. Molecular products and radicals from pyrolysis of lignin. Environ. Sci. Technol. 2012, 46 (23), 12994–13001. 10.1021/es302942c. 23131040

[ref30] Schuler J.; Hornung U.; Kruse A.; Dahmen N.; Sauer J. Hydrothermal Liquefaction of Lignin. J. Biomaterials Nanobiotechnol. 2017, 08 (01), 96–108. 10.4236/jbnb.2017.81007.

[ref31] Schuler J.; Hornung U.; Dahmen N.; Sauer J. Lignin from bark as a resource for aromatics production by hydrothermal liquefaction. GCB Bioenergy 2019, 11 (1), 218–229. 10.1111/gcbb.12562.

[ref32] Schmiedl D.; Böringer S.; Tübke B.; Liitiä T.; Rovio S.; Tamminen T.; Rencoret J.; Gutiérrez A.; del Rio J. C. Kraft lignin depolymerisation by base catalysed degradation-Effect of process parameters on conversion degree, structural features of BCD fractions and their. NWBC 2015: The 6th Nordig Wood Biorefinery Conference: Helsinki, Finland, 20–22 October, 2015; Hytönen E., Ed.; VTT Technical Research Centre of Finland Ltd, 2015; Vol. 233.

[ref33] Doassans-Carrère N.; Ferrasse J.-H.; Boutin O.; Mauviel G.; Lédé J. Comparative Study of Biomass Fast Pyrolysis and Direct Liquefaction for Bio-Oils Production: Products Yield and Characterizations. Energy Fuels 2014, 28 (8), 5103–5111. 10.1021/ef500641c.

[ref34] Kruse A.; Dahmen N. Water - A magic solvent for biomass conversion. J. Supercrit. Fluids 2015, 96, 36–45. 10.1016/j.supflu.2014.09.038.

[ref35] Akiya N.; Savage P. E. Roles of water for chemical reactions in high-temperature water. Chem. Rev. 2002, 102 (8), 2725–2750. 10.1021/cr000668w. 12175266

[ref36] Hunter S. E.; Savage P. E. Recent advances in acid- and base-catalyzed organic synthesis in high-temperature liquid water. Chem. Eng. Sci. 2004, 59 (22–23), 4903–4909. 10.1016/j.ces.2004.09.009.

[ref37] Franck E. U.; Rosenzweig S.; Christoforakos M. Calculation of the Dielectric Constant of Water to 1000°C and Very High Pressures. Ber. Bunsenges. Phys. Chem. 1990, 94 (2), 199–203. 10.1002/bbpc.19900940219.

[ref38] Belkheiri T.; Andersson S.-I.; Mattsson C.; Olausson L.; Theliander H.; Vamling L. Hydrothermal Liquefaction of Kraft Lignin in Subcritical Water: Influence of Phenol as Capping Agent. Energy Fuels 2018, 32 (5), 5923–5932. 10.1021/acs.energyfuels.8b00068.

[ref39] Breunig M.; Gebhart P.; Hornung U.; Kruse A.; Dinjus E. Direct liquefaction of lignin and lignin rich biomasses by heterogenic catalytic hydrogenolysis. Biomass Bioenergy 2018, 111, 352–360. 10.1016/j.biombioe.2017.06.001.

[ref40] Forchheim D.; Hornung U.; Kempe P.; Kruse A.; Steinbach D. Influence of RANEY Nickel on the Formation of Intermediates in the Degradation of Lignin. Int. J. Chem. Eng. 2012, 2012 (4), 1–8. 10.1155/2012/589749.

[ref41] Cheng S.; Wilks C.; Yuan Z.; Leitch M.; Xu C. Hydrothermal degradation of alkali lignin to bio-phenolic compounds in sub/supercritical ethanol and water-ethanol co-solvent. Polym. Degrad. Stab. 2012, 97 (6), 839–848. 10.1016/j.polymdegradstab.2012.03.044.

[ref42] Forchheim D.; Hornung U.; Kruse A.; Sutter T. Kinetic Modelling of Hydrothermal Lignin Depolymerisation. Waste Biomass Valorization 2014, 5 (6), 985–994. 10.1007/s12649-014-9307-6.

[ref43] Gasson J. R.; Forchheim D.; Sutter T.; Hornung U.; Kruse A.; Barth T. Modeling the Lignin Degradation Kinetics in an Ethanol/Formic Acid Solvolysis Approach. Part 1. Kinetic Model Development. Ind. Eng. Chem. Res. 2012, 51 (32), 10595–10606. 10.1021/ie301487v.

[ref44] Toledano A.; Serrano L.; Labidi J. Improving base catalyzed lignin depolymerization by avoiding lignin repolymerization. Fuel 2014, 116, 617–624. 10.1016/j.fuel.2013.08.071.

[ref45] Wahyudiono; Sasaki M.; Goto M. Recovery of phenolic compounds through the decomposition of lignin in near and supercritical water. Chem. Eng. Process 2008, 47 (9–10), 1609–1619. 10.1016/j.cep.2007.09.001.

[ref46] Yong T. L.-K.; Matsumura Y. Reaction Kinetics of the Lignin Conversion in Supercritical Water. Ind. Eng. Chem. Res. 2012, 51 (37), 11975–11988. 10.1021/ie300921d.

[ref47] Yong T. L.-K.; Matsumura Y. Kinetic Analysis of Lignin Hydrothermal Conversion in Sub- and Supercritical Water. Ind. Eng. Chem. Res. 2013, 52 (16), 5626–5639. 10.1021/ie400600x.

[ref48] Lappalainen J.; Baudouin D.; Hornung U.; Schuler J.; Melin K.; Bjelić S.; Vogel F.; Konttinen J.; Joronen T. Sub- and Supercritical Water Liquefaction of Kraft Lignin and Black Liquor Derived Lignin. Energies 2020, 13 (13), 3309 10.3390/en13133309.

[ref49] Orebom A.; Verendel J. J.; Samec J. S. M. High Yields of Bio Oils from Hydrothermal Processing of Thin Black Liquor without the Use of Catalysts or Capping Agents. ACS Omega 2018, 3 (6), 6757–6763. 10.1021/acsomega.8b00854. 31458848 PMC6644617

[ref50] Cardoso M.; de Oliveira É. D.; Passos M. L. Chemical composition and physical properties of black liquors and their effects on liquor recovery operation in Brazilian pulp mills. Fuel 2009, 88 (4), 756–763. 10.1016/j.fuel.2008.10.016.

[ref51] Argyropoulos D. S. 31P NMR in wood chemistry: A review of recent progress. Res. Chem. Intermed. 1995, 21 (3–5), 373–395. 10.1007/BF03052265.

[ref52] Kim H.; Ralph J.; Lu F.; Ralph S. A.; Boudet A. M.; MacKay J. J.; Sederoff R. R.; Ito T.; Kawai S.; Ohashi H.; et al. NMR analysis of lignins in CAD-deficient plants. Part 1. Incorporation of hydroxycinnamaldehydes and hydroxybenzaldehydes into lignins. Org. Biomol. Chem. 2003, 1 (2), 268–281. 10.1039/b209686b. 12929422

[ref53] Terashima N.; Atalla R. H.; Vanderhart D. L. Solid state NMR spectroscopy of specifically 13C-enriched lignin in wheat straw from coniferin. Phytochemistry 1997, 46 (5), 863–870. 10.1016/S0031-9422(97)00359-2.

[ref54] Pu Y.; Cao S.; Ragauskas A. J. Application of quantitative 31P NMR in biomass lignin and biofuel precursors characterization. Energy Environ. Sci. 2011, 4 (9), 3154 10.1039/c1ee01201k.

[ref55] Bartolomei E.; Brech Y. L.; Gadiou R.; Bertaud F.; Leclerc S.; Vidal L.; Meins J. M. L.; Dufour A. Depolymerization of Technical Lignins in Supercritical Ethanol: Effects of Lignin Structure and Catalyst. Energy Fuels 2021, 35 (21), 17769–17783. 10.1021/acs.energyfuels.1c02704.

[ref56] Le Brech Y.; Raya J.; Delmotte L.; Brosse N.; Gadiou R.; Dufour A. Characterization of biomass char formation investigated by advanced solid state NMR. Carbon 2016, 108, 165–177. 10.1016/j.carbon.2016.06.033.

[ref57] Crestini C.; Lange H.; Sette M.; Argyropoulos D. S. On the structure of softwood kraft lignin. Green Chem. 2017, 19 (17), 4104–4121. 10.1039/C7GC01812F.

[ref58] Karlsson M.; Romson J.; Elder T.; Emmer Å.; Lawoko M. Lignin Structure and Reactivity in the Organosolv Process Studied by NMR Spectroscopy, Mass Spectrometry, and Density Functional Theory. Biomacromolecules 2023, 24 (5), 2314–2326. 10.1021/acs.biomac.3c00186. 37078866 PMC10170516

[ref59] Springer. VDI-Wärmeatlas; Springer Berlin Heidelberg, 2013.

[ref60] Korntner P.; Sumerskii I.; Bacher M.; Rosenau T.; Potthast A. Characterization of technical lignins by NMR spectroscopy: optimization of functional group analysis by 31 P NMR spectroscopy. Holzforschung 2015, 69 (6), 807–814. 10.1515/hf-2014-0281.

[ref61] Belkheiri T.; Mattsson C.; Andersson S.-I.; Olausson L.; Åmand L.-E.; Theliander H.; Vamling L. Effect of pH on Kraft Lignin Depolymerisation in Subcritical Water. Energy Fuels 2016, 30 (6), 4916–4924. 10.1021/acs.energyfuels.6b00462.

[ref62] Belkheiri T.; Andersson S.-I.; Mattsson C.; Olausson L.; Theliander H.; Vamling L. Hydrothermal liquefaction of kraft lignin in sub-critical water: the influence of the sodium and potassium fraction. Biomass Convers. Biorefin. 2018, 8 (3), 585–595. 10.1007/s13399-018-0307-9.

[ref63] Nimmanwudipong T.; Runnebaum R. C.; Block D. E.; Gates B. C. Catalytic Conversion of Guaiacol Catalyzed by Platinum Supported on Alumina: Reaction Network Including Hydrodeoxygenation Reactions. Energy Fuels 2011, 25 (8), 3417–3427. 10.1021/ef200803d.

[ref64] Ishikawa M.; Tamura M.; Nakagawa Y.; Tomishige K. Demethoxylation of guaiacol and methoxybenzenes over carbon-supported Ru-Mn catalyst. Appl. Catal., B 2016, 182, 193–203. 10.1016/j.apcatb.2015.09.021.

[ref65] Zhu X.; Lobban L. L.; Mallinson R. G.; Resasco D. E. Bifunctional transalkylation and hydrodeoxygenation of anisole over a Pt/HBeta catalyst. J. Catal. 2011, 281 (1), 21–29. 10.1016/j.jcat.2011.03.030.

[ref66] Kruse A. Supercritical water gasification. Biofuels, Bioprod. Biorefin. 2008, 2 (5), 415–437. 10.1002/bbb.93.

[ref67] Ma H.; Li T.; Wu S.; Zhang X. Demethylation of a methoxy group to inhibit repolymerization during alkaline lignin pyrolysis. Fuel 2021, 286, 119394 10.1016/j.fuel.2020.119394.

[ref68] Wang C.; Fan Y.; Hornung U.; Zhu W.; Dahmen N. Char and tar formation during hydrothermal treatment of sewage sludge in subcritical and supercritical water: Effect of organic matter composition and experiments with model compounds. J. Clean. Prod. 2020, 242, 118586 10.1016/j.jclepro.2019.118586.

[ref69] Towfighi J.; Sadrameli M.; Niaei A. Coke Formation Mechanisms and Coke Inhibiting Methods in Pyrolysis Furnaces. J. Chem. Eng. 2002, 35 (10), 923–937. 10.1252/jcej.35.923.

[ref70] Asafu-Adjaye O. A.; Street J.; Bansode A.; Auad M. L.; Peresin M. S.; Adhikari S.; Liles T.; Via B. K. Fast Pyrolysis Bio-Oil-Based Epoxy as an Adhesive in Oriented Strand Board Production. Polymers 2022, 14 (6), 1244 10.3390/polym14061244. 35335574 PMC8950851

[ref71] Li M.; Yoo C. G.; Pu Y.; Ragauskas A. J. 31 P NMR Chemical Shifts of Solvents and Products Impurities in Biomass Pretreatments. ACS Sustain. Chem. Eng. 2018, 6 (1), 1265–1270. 10.1021/acssuschemeng.7b03602.

[ref72] Zhao J.; Xiuwen W.; Hu J.; Liu Q.; Shen D.; Xiao R. Thermal degradation of softwood lignin and hardwood lignin by TG-FTIR and Py-GC/MS. Polym. Degrad. Stab. 2014, 108, 133–138. 10.1016/j.polymdegradstab.2014.06.006.

[ref73] Pipitone G.; Zoppi G.; Bocchini S.; Rizzo A. M.; Chiaramonti D.; Pirone R.; Bensaid S. Aqueous phase reforming of the residual waters derived from lignin-rich hydrothermal liquefaction: investigation of representative organic compounds and actual biorefinery streams. Catal. Today 2020, 345, 237–250. 10.1016/j.cattod.2019.09.040.

[ref74] Pola L.; Collado S.; Wörner M.; Hornung U.; Díaz M. Eutectic solvents for the valorisation of the aqueous phase from hydrothermally liquefied black liquor. J. Environ. Chem. Eng. 2023, 11 (5), 111040 10.1016/j.jece.2023.111040. 37544524

