# Supplementary material for: From Pulp to Aromatic Products—Reaction Pathways of Lignin Depolymerization
Source: Energy Fuels. 2024 Mar 13;38(7):6020–35. doi: 10.1021/acs.energyfuels.3c04509 (PMC11000225; doi:10.1021/acs.energyfuels.3c04509)
Supplement: Supplementary file 1 — ef3c04509_si_001.docx [file ef3c04509_si_001.docx]

**Supporting Information**

**From Pulp to Aromatic Products – Reaction Pathways of Lignin Depolymerization**

Maximilian Wörner (maximilian.woerner@kit.edu)*, Alexandra Barsuhn (unebf@student.kit.edu), Thomas Zevaco (thomas.zevaco@kit.edu), Ursel Hornung (ursel.hornung@kit.edu), Nicolaus Dahmen (nicolaus.dahmen@kit.edu)

^†^Karlsruhe Institute of Technology (KIT), Institute of Catalysis Research and Development (IKFT), Hermann-von-Helmholtz-Platz 1, 76344 Eggenstein-Leopoldshafen, Germany

**Table S1**. Weighed-in masses of salts used for preparation of 100 mL of salt solution

|  | **Weighed-in mass / g** |
| --- | --- |
| **Na_2_CO_3_** | 1.781 |
| **K_2_CO_3_** | 0.275 |
| **Na_2_SO_3_** | 0.132 |
| **Na_2_SO_4_** | 0.109 |
| **Na_2_S_2_O_3_** | 0.194 |
| **Na_2_S * 9 H_2_O** | 2.363 |

**Figure S1**. Micro autoclave station to open and close the autoclaves; dashed line shows filling process, solid line shows opening process; a) container where the micro autoclave is placed in, b) gas trap with septum for taking gas samples; c) manometer to measure the pressure in the system after opening the reactor; d) outlet for purging the system

**Table S2**. Volumes of black liquor used at specific reaction temperatures *T*_R_

| ***T*_R_** | 250 – 275 °C | 300 – 350 °C | 375 °C | 400 °C |
| --- | --- | --- | --- | --- |
| ***V*_Feed_** | 17.5 mL | 15 mL | 12.5 mL | 5 mL |

**Calculation of the carbon mass in the gas phase**

With the help of a manometer (PI in **Figure S1**), the pressure *p* could be determined. Since this pressure describes the entire system instead of only the micro autoclave, a correction is necessary for later calculations of the resulting gas phase. The correlation to the pressure in the reactor $p_{corr}$ was determined before by filling the autoclaves with nitrogen up to different pressures and releasing the gas afterwards into the micro autoclave station (see SI **Table S3**).

| $p_{corr}= \frac{p}{0,083}$ | (S1) |
| --- | --- |

**Table S3**. Data for calculation of the used coefficient in **equation S1** to calculate $p_{corr}$. Autoclaves are filled with N_2_ up to the pressure $p_{Autoclave}$. After opening the autoclave in the autoclave station (**Figure S1**) $p_{System}$.is shown on the manometer. $p_{Autoclave}$ was in the range from 0 to 2 bar for all the experiments done for this study.

|  | $\boldsymbol{p}_{\boldsymbol{Autoclave}}$ **/ bar** | $\boldsymbol{p}_{\boldsymbol{System}}$ **/ bar** | **Calculated coefficient** |
| --- | --- | --- | --- |
|  | 5 | 0.4 | 0.08 |
|  | 10 | 0.8 | 0.08 |
|  | 15 | 1.3 | 0.87 |
|  | 20 | 1.7 | 0.085 |
| **Mean coefficient** |  |  | 0.083 |

**Equation S2** is used to determine the amount of substance in each gas component *n*_i_. *V* is the free volume in the micro autoclave. The room temperature *T*_Room_ = 296.15 K is used as the temperature and *R* is the universal gas constant. The measured volume fraction of the gas compound via GC-FID/TCD is inserted as $\varphi_{i}$*.* From this, the total mass of carbon *m*_c,gas_, which is relevant for the carbon mass balance, can be calculated in the next step (**Equation S3)** For this, the number of carbon atoms per molecule and the molecular weight of carbon *M*_c_ are required. Table S7 lists all the detectable gas compounds with our setup.

| $n_{i}= \frac{p_{corr}*V*\frac{\varphi_{i}}{100}}{R*T_{room}}$ | (S2) |
| --- | --- |
|  |  |
| $m_{c,g}=\left( \sum n_{i}*v_{i} \right)*M_{C}$ | (S3) |

**Table S4**. Gas compounds which are detectable with the used GC setup (TCD and FID)

| **Permanent gases (TCD)** | **Hydrocarbons (FID)** |
| --- | --- |
| H_2_ | CH_4_ |
| CO | C_2_H_4_ |
| CO_2_ | C_2_H_6_ |
| O_2_ | C_3_H_6_ |
| N_2_ | C_3_H_8_ |
|  | n-C_4_H_10_ |
|  | iso-C_4_H_10_ |

**Table S5**. Experimentally determined distribution coefficients for different monocyclic phenolic compounds when using the described extraction procedure

| **Component *i*** | **Distribution coefficient *K*_i_** |
| --- | --- |
| Phenol | 0.95 |
| Guaiacol | 0.92 |
| Catechol | 0.82 |
| 3-Methoxycatechol | 0.7 |
| 3-Methylcatechol | 0.83 |
| 4-Methylcatechol | 0.7 |
| Syringol | 0.75 |
| Syringaldehyde | 0.67 |
| Acetosyringon | 0.65 |

**Detailed information about the ^13^C NMR analysis**

The calibration and optimization of the recording parameters for ^13^C (among others, contact time for the CP/MAS) have been optimized using adamantane and glycine. All the samples were beforehand grinded to give homogeneous, finely powdered materials. The samples were filed in proprietary 3,2 mm Rotors (zirconia with Vespel/PEEK caps). The spectra were recorded at a spinning rate of 15 KHz.

The ^13^C single pulse spectra were recorded using the standard pulses sequences single_pulse_dec_solid.jxp cpmas_dipolar.jxp and cpmas_dipolar_dephase.jxp with a ^1^H 90°pulse length of 2.7 μs, an optimized contact time of 1 ms for CP/MAS (checked using a CT array from 0.5 to 2 ms) , a relaxation delay of 5 s, a spectral width of 500 ppm (x_sweep); 4096 scans. Chemical shifts, δ, were calibrated relatively to the adamantane signals (C_10_H_16_: δ =xx and yy ppm) as external standard.

The measurements are conducted using the proprietary software of Jeol Delta 5.3.3., the pulse sequences used belong to the standard Delta library and were optimized when necessary. The evaluation and plotting of the spectra were performed using Delta 5.3.3 and the MNova 10 NMR software package (https://mestrelab.com/software/mnova/nmr/).
